# CeO_2_ Frustrated Lewis Pairs Improving CO_2_ and CH_3_OH Conversion to Monomethylcarbonate

**DOI:** 10.1021/acsami.2c22122

**Published:** 2023-03-14

**Authors:** Davide Salusso, Giorgio Grillo, Maela Manzoli, Matteo Signorile, Spyridon Zafeiratos, Mathias Barreau, Alessandro Damin, Valentina Crocellà, Giancarlo Cravotto, Silvia Bordiga

**Affiliations:** † Department of Chemistry, University of Turin, 10125 Turin, Italy; ○ NIS Center, University of Turin, 10125 Turin, Italy; ⊗ INSTM Reference Center, University of Turin, 10125 Turin, Italy; ‡ European Synchrotron Radiation Facility, CS 40220, Cedex 9 38043 Grenoble, France; § Department of Drug Science and Technology, University of Turin, 10125 Turin, Italy; ∥ Institut de Chimie et Procédés pour L’Energie, L’Environnement et La Santé, UMR 7515 CNRS-UdS, 25 Rue Becquerel, 67087 Strasbourg, France

**Keywords:** CeO_2_, frustrated Lewis pair, CO_2_ activation, dimethyl carbonate, monomethylcarbonate

## Abstract

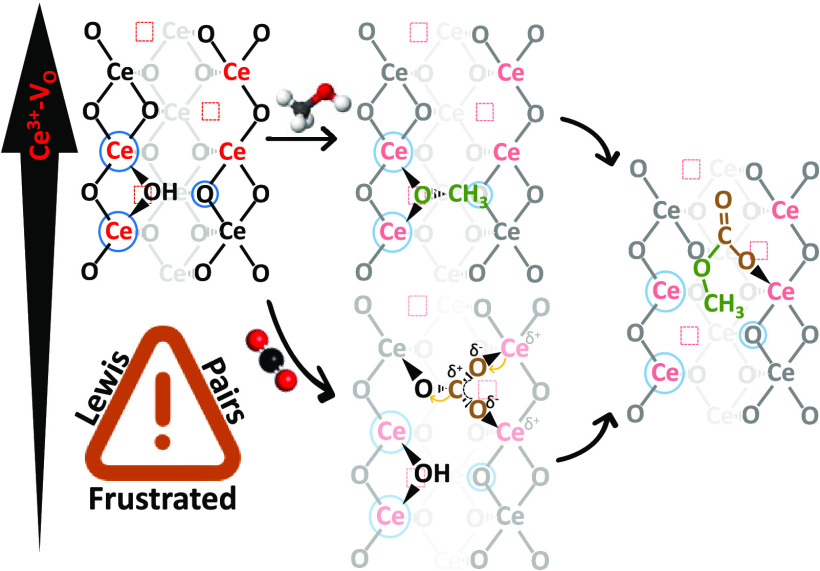

Frustrated Lewis pairs (FLPs), discovered in the last few decades for homogeneous catalysts and in the last few years also for heterogeneous catalysts, are stimulating the scientific community’s interest for their potential in small-molecule activation. Nevertheless, how an FLP activates stable molecules such as CO_2_ is still undefined. Through a careful spectroscopic study, we here report the formation of FLPs over a highly defective CeO_2_ sample prepared by microwave-assisted synthesis. Carbon dioxide activation over FLP is shown to occur through a bidentate carbonate bridging the FLP and implying a Ce^3+^-to-CO_2_ charge transfer, thus enhancing its activation. Carbon dioxide reaction with methanol to form monomethylcarbonate is here employed to demonstrate active roles of FLP and, eventually, to propose a reaction mechanism clarifying the role of Ce^3+^ and oxygen vacancies.

## 1 Introduction

Frustrated Lewis pairs (FLPs) consist of a Lewis acid (LA) and a Lewis base (LB) with sterically hindered ligands that prevent these species from neutralizing each other.^1,2^ Historically discovered in 1942^3^ and first named in 2010,^4^ FLPs were first limited to homogeneous catalysis but accessed recently for heterogeneous catalysis.^1,2,5−8^ In particular, CeO_2_ has been shown to form FLPs between two adjacent Ce^3+^ (LAs) and an O^2–^ (LB) separated by an oxygen vacancy at a distance of ∼4 Å.^9,10^ This particular condition was shown to occur only when the surface Ce^3+^ concentration (>30%) ensured clustering of Ce^3+^–V_O_–Ce^3+^ over CeO_2_(101) planes.^11^ CeO_2_ FLPs improved the reactivity of alkenes and alkynes,^9,10^ syngas,^12^ and recently CO_2._^13,14^ Considering the latter case, CO_2_ activation over heterogeneous catalysts occurs through an acid–base interaction with the catalyst surface, inducing the bending of the CO_2_ double bond and making the C atom more electrophilic.^15^ Zhang and co-workers predicted as CeO_2_ FLPs could activate CO_2_ through formation of bidentate carbonate, hence improving CO_2_ conversion toward olefins and cyclic carbonates.^14^ Nevertheless, the mechanism on how the FLP site activates CO_2_ remains unclear. Indeed, the presence and activity of FLP are very difficult to be observed since it mainly consists of missing oxygens on a catalyst surface well-known for its properties of oxygen storage and mobility.^16^ For this reason, an improved CO_2_ conversion at high temperature over Ce^3+^-rich CeO_2_ to FLP is not straightforward. To disclose details on the CO_2_/FLP interaction, we investigated the monomethylcarbonate (MMC) formation reaction from CO_2_ and CH_3_OH. MMC is the intermediate leading to the formation of dimethyl carbonate (DMC) through the direct reaction of CO_2_ and CH_3_OH, an environmentally friendly process due to its potential toward CO_2_ mitigation.^17−22^ This is indeed an ideal case study for studying CO_2_/FLP interaction since it implies CO_2_ whole incorporation in a new molecule (CH_3_O(CO_2_)-) at a moderate temperature (≈150 °C), which prevents CeO_2_ oxygen mobility, i.e., reducing the surface-to-bulk FLP mobility. Moreover, MMC formation represents the most challenging reaction step since it implies CO_2_ activation. As first formulated by Jung and Bell,^23^ the reaction usually exploits both acid and base Lewis sites over an amphoteric catalyst such as ZrO_2_, CeO_2_, or their solid solutions. Several studies showed that the presence of oxygen vacancies, induced by Zr doping of CeO_2_, could promote CO_2_ activation through carbonate formation and directly improving DMC production.^24−28^ Considering that pure CeO_2_ oxygen vacancies formed over (110) planes were predicted to form more easily bidentate carbonates however, the presence of Ce^3+^ in CeO_2_ was also directly related to catalyst deactivation.^29−31^ Recently, Li and co-workers reported that FLP at CeO_2_ improved DMC formation.^32^ The catalytic tests indicated a higher DMC yield for the highly reduced ceria (Ce^3+^ ≈ 19%), while density functional theory (DFT) modeling predicted that the Ce^3+^-to-CO_2_ charge transfer decreased the CO_2_ activation energy. To further investigate and clarify the role of FLP toward CO_2_ and CH_3_OH activations, we here compared MMC formation over four CeO_2_ samples with modulated defectivity and Ce^3+^ concentration, aiming to differentiate CO_2_ conversion between defects and FLP presence. Microwave-assisted sol–gel synthesis was employed to prepare defective CeO_2_ samples containing a relevant amount of Ce_2_O_3_ and Ce_6_O_11_, allowing ca. 35% of surface Ce^3+^ at a moderate temperature (150 °C). FLP formation was confirmed through X-ray photoelectron spectroscopy (XPS) as well as Raman and infrared (IR) spectroscopies. CO_2_ and CH_3_OH activation over Ce^4+^/Ce^3+^ and FLP sites was monitored by XPS, IR, and ultraviolet–visible (UV–vis) spectroscopies. Eventually, a reaction mechanism involving CO_2_/CH_3_OH and FLP sites is hypothesized.

## 2 Materials and Methods

### 2.1 Microwave-Assisted Preparation of Ceria Catalysts

CeO_2_ catalysts were prepared by microwave (MW)-assisted sol–gel synthesis adapted from a conventional protocol.^33,34^ Briefly, 1.0 g of (NH_4_)_2_[Ce(NO_3_)_6_] was dissolved in 14.5 mL of a solution containing an excess of urea (approximately 0.12 g/mL). The mixture was placed in a glass vial and heated in a monomodal MW reactor (Anton Paar Monowave Microwave 300) up to 120 °C for 1 h. The selected irradiation protocol was set to work with free power (max. 850 W) to reach the working temperature in the fastest way, maintaining 800 rpm of stirring. Urea degradation to ammonia decreased the pH solution, allowing CeO_2_ precipitation. The formed precipitate was recovered by means of centrifugation (1 min, 26,000 rpm) and dried for 24 h at 100 °C. The obtained yellow powder was then divided into two batches, named MW(100) and MW(650), which were dried under air for 8 h at 100 °C or calcined at 650 °C for the same time, respectively.

To investigate the possible effect of MW on the catalyst properties, a reference material was prepared by conventional sol–gel synthesis, referred to as conv(650).^33,34^ In this case, the 14.5 mL mixture containing the (NH_4_)_2_[Ce (NO_3_)_6_] precursor (1.0 g) and the urea excess was refluxed under stirring (approx. 100 °C, 600 rpm) for 8 h; then, the precipitate was washed with boiling deionized water, dried at 100 °C overnight, and finally calcined at 650 °C for 8 h.

### 2.2 Catalyst Characterization

Specific surface areas (SSAs) of CeO_2_ samples were determined by applying the Brunauer–Emmett–Teller (BET) method to the absorption/desorption isotherms of N_2_ at –196 °C obtained with a Micromeritics ASAP 2010 physisorption analyzer. The adsorption/desorption isotherms were measured over a wide range of relative pressures (10^–3^ < *p*/*p*_0_ < 1). Pore size distribution was calculated applying the NL-DFT (N_2_, –196 °C, carbon, slit pores model) method. All of the samples underwent an activation step to remove physisorbed species from the surface while avoiding irreversible changes of the surface or the solid structure. Each sample was studied after outgassing under vacuum at 400 °C (heating ramp of 5 °C min^–1^) for 5 h (residual pressure as low as 10^–4^ mbar).

Powder X-ray diffractograms (PXRDs) were measured with a PW3050/60 X’Pert PRO MPD diffractometer from PANalytical working in Bragg–Brentano geometry, equipped with a Cu Kα_1/2_ X-ray source. Catalysts were measured at room temperature with a spinning zero background Si crystal sample holder, in the 10–100° 2θ range. Lattice parameters, peak intensity, and profile were refined using the Rietveld method implemented in Fullprof software.^35^ To prevent air contamination, PXRDs after oxidation and reduction treatments were collected in the transmission mode on sealed glass capillaries (ø = 0.3 mm) at BM31 Beamline of the European Synchrotron Radiation Facility (ESRF) using monochromatic 46 KeV (≈0.270 Å) incident radiation.

High-resolution transmission electron microscopy (HR-TEM) was employed to achieve morphological and structural information of all of the CeO_2_ samples with a side entry Jeol (Akishima, Tokyo, Japan) JEM 3010 UHR (300 kV, LaB_6_ filament). The samples were deposited on a Cu grid coated with a lacey carbon film. All digital micrographs were acquired by an UltraScan 1000 camera, and the images were processed by Gatan digital micrograph (Pleasanton, CA). Particle size distributions of MW(100) and MW(650) catalysts were obtained by counting a statistically representative number of particles for each sample (>350 for MW(100), >250 in the case of MW(650)). The mean particle diameter (*d*_m_) was calculated as
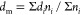
where *n_i_* is the number of particles of diameter *d_i_*.

Differences in the CeO_2_ structure potentially arising from the preparation methods were investigated by means of a statistical analysis of the interplanar spacings measured on the Fourier transform (FT) of all high-resolution transmission electron microscopy (HR-TEM) images collected for each sample. To build the spacing distribution, expressed as percentage (%), a statistically representative number of measured spacings for each sample (260 for MW(100), 335 in the case of MW(650), and 273 for conv(650)) was considered.

Fourier transform IR spectra were collected in the transmission mode using a Bruker Vertex 70 spectrometer equipped with an MCT detector in the 4000–600 cm^–1^ range with 2 cm^–1^ resolution. Samples were pressed in self-supporting pellets (ca. 10 mg/cm^2^) and placed in quartz IR cells suitable for thermal treatments in a controlled atmosphere and for spectra recording at room temperature (RT) and nominal liquid nitrogen temperature (LNT). Before IR measurements, catalysts underwent an activation meant to clean the catalyst surface leaving an oxidized/reduced state. In both cases, the followed protocol consisted of different steps listed here: (i) outgassing and heating the catalyst at 5 °C/min from RT to 150 °C under vacuum, (ii) heating from 150 to 400 °C (5 °C/min) under static 100 mbar of O_2_ to prevent CeO_2_ self-reduction via oxygen depletion from the surface, (iii) holding at 400 °C for 30′ changing the O_2_ atmosphere 3 times, and (iv) cooling under O_2_ until 150 °C and then evacuating. The reduced catalyst (hereafter referred to as MW(100)-red) was prepared in situ starting from the oxidized catalyst (hereafter referred to as MW(100)) just before each characterization measurement. Briefly, a pellet of MW(100) after its activation was kept in the quartz IR cell where it was heated under vacuum to 150 °C for 60 min, employing a dedicated homemade setup for activation of samples in a controlled atmosphere, i.e., avoiding any exposure to air. The pellet was then exposed to pure H_2_ (static, 100 mbar) at 150 °C for 30′, changing the H_2_ atmosphere three times. The sample was then evacuated at 150 °C for 30 min and cooled down to room temperature under vacuum.

CO, CO_2_, and CH_3_OH interactions were investigated by exposing the cleaned pellet to the molecules, with pressures of 3, 100, and 40 mbar, respectively. Spectra were treated using Bruker OPUS spectroscopy software, while CO fit was conducted using CasaXPS software, by applying a linear background and describing bands with a pure Lorentzian function.

Diffuse reflectance UV–vis spectra were collected in a Varian Cary 5000 spectrophotometer, equipped with an integrating sphere with the inner surface coated by Spectralon (the same material used as the white reference). The powders were placed in a quartz bulb cell, allowing thermal treatments. Pretreatments and CO_2_ interaction were performed in the same way as for the IR measurements.

Quasi-in situ X-ray photoelectron spectroscopy (XPS) measurements were carried out in an ultrahigh vacuum (UHV) spectrometer as described elsewhere.^36^ The spectrometer is equipped with a variable-pressure reactor allowing thermal/gas treatments of the catalyst in a controlled atmosphere and consequent transfer to the XPS analysis chamber without exposing it to air. An online differentially pumped mass spectrometer was installed in the reactor to verify potential CO production. The Al Kα line (1486.6 eV) of a dual-anode X-ray source was used as incident radiation. Survey and high-resolution spectra were recorded in constant-pass energy modes (44 and 22 eV, respectively). The C 1s peak of adventitious carbon was used as the reference for the binding energies. Catalyst activation followed the same procedure described above for IR measurements, with the only exception being operation in a flow (not static) gas atmosphere. Moreover, CO_2_ and CH_3_OH interactions with the MW(100)-red surface have been investigated at 30 and 150 °C. To minimize beam damage (see Supporting Information Section 2.1), Ce 3d peaks were measured as the first region with 30′ time/scan. Spectra fitting was performed with CasaXPS software. Due to the complex peak shape, fitting of the Ce 3d region is not straightforward. In this work, Ce 3d peak fitting was conducted applying thoughtful constraints to the peak position, full width at half-maximum, and the area following Paparazzo guidelines.^37^ In particular, six peaks were used for Ce^(IV)^O_2_ and four for Ce_2_^(III)^O_3_, named ν, ν″, ν‴, *u*, *u*″, *u*‴ and ν^0^, ν′, *u*^0^, *u*′, respectively, constraining their positions to a fixed spin–orbit splitting (Δ_s-o_) of 18.5 eV. Gaussian–Lorentzian (50:50) functions were employed for describing the peak shape; the full width at half-maximum was fixed between spin–orbit couples, while the peak’s area was constrained with to respect the intercomponent peak intensity ratio, i.e., *I*_ν_^n^/*I_u_^n^* = 1.5 ± 0.1 (*n* = 0, ′, ″, ‴). Background was described using the spline Shirley function, while the Ce^3+^/Ce^4+^ ratio was calculated as
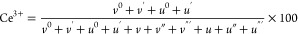
The O 1s region was fitted with two components (pseudo-Voight band shape) using the linear background.

Raman spectra were recorded at room temperature using (i) a Renishaw micro-Raman System 1000 with He/Cd laser (Kimmon) emitting at 325 nm and (ii) a Renishaw micro-Raman System 1000 with Ar^+^ laser (Spectra Physics) with 514 nm emission. Spectra were collected on self-supporting pellets contained in a homemade cell composed of a suprasil-quartz cuvette (Hellma, 2 mm optic path) sealed to a quartz tube, allowing thermal treatments. Samples were pretreated following the same activation procedure as described for the IR spectra.

## 3 Results and Discussion

### 3.1 Textural, Structural, and Morphological Properties of Catalysts

The CeO_2_ catalyst prepared by conventional synthesis (conv(650), Section 2.1) presented a low SSA (8 m^2^/g Table S1), while PXRD analysis revealed the presence of large crystallites in the cubic phase (Figure S1c, JCPDS file number 34-394). On the contrary, MW-assisted synthesis (MW(100) and MW(650)) allowed the precipitation of CeO_2_ in the cubic polymorph with much smaller crystallites (Figure S1a) and higher SSA (43 vs 75 m^2^/g) (Figure S2).

**Figure 1:**
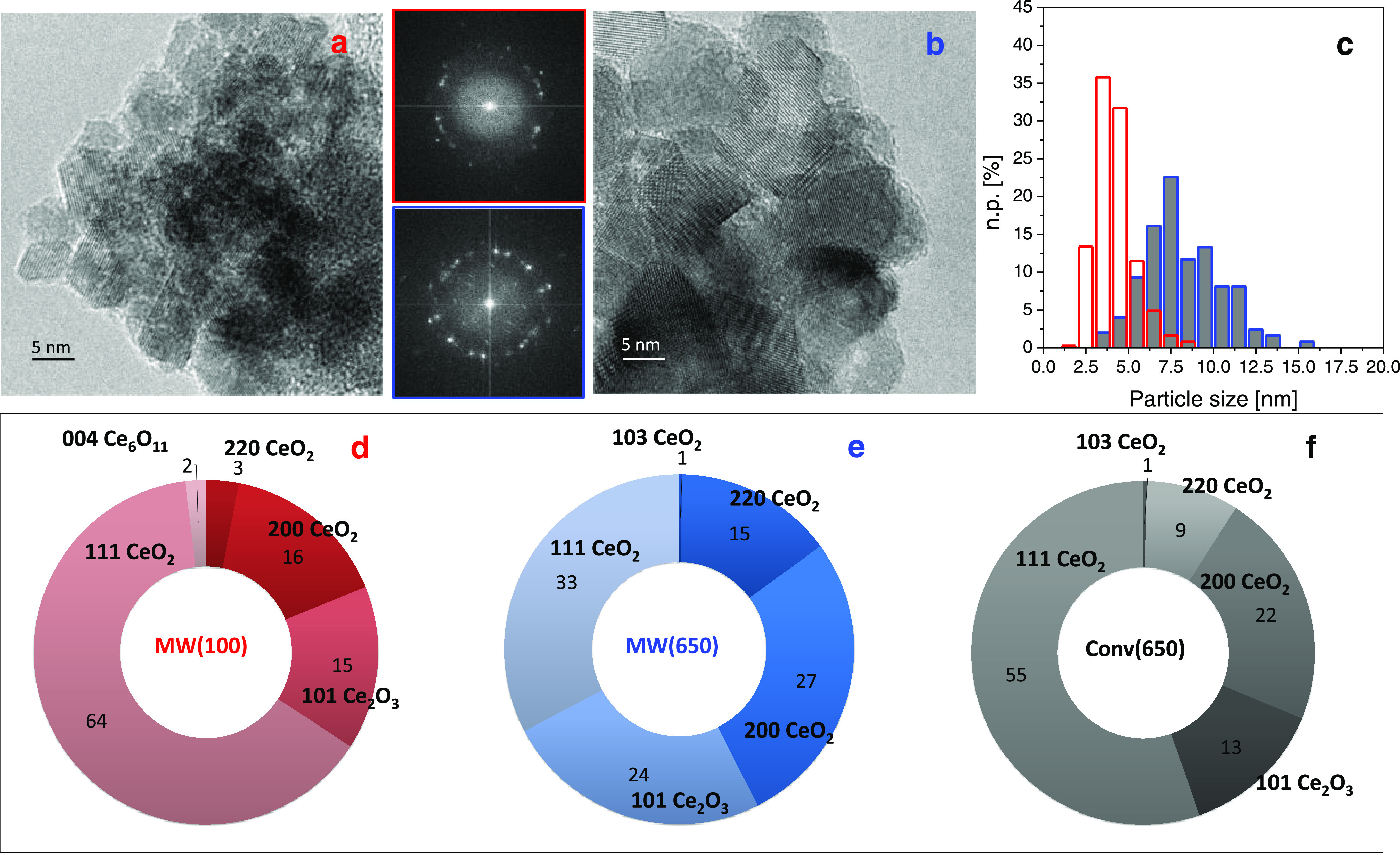
HR-TEM representative images of (a) MW(100) and (b) MW(650) and (c) corresponding particle size distributions (red and blue, respectively). Insets: FT of the images shown in (a) (red box) and (b) (blue box). Statistical distribution of the interplanar spacings, expressed as percentage (%), for MW(100) (d), MW(650) (e), and conv(650) (f). Instrumental magnification: 500,000×.

The results of the HR-TEM analyses carried out on both MW(100) and MW(650) catalysts are summarized in Figure 1, whereas the characterization of the conv(650) reference sample is reported in SI Section 1 (Figure S3).

Overall, the MW(100) is composed of small crystalline nanoparticles, with a square shape (Figure 1a), and homogeneous in size as revealed by the particle size distribution (Figure 1, red columns), where a large fraction of nanoparticles (>65%) has size between 2.5 and 5.0 nm, which results in a mean particle diameter *d*_m_ of 4.1 ± 1.2 nm. The subsequent calcination at 650 °C (Figure 1b) induced an increase of the particle size (Figure 1c, blue columns), resulting in a *d*_m_ = 8.1 ± 2.2 nm. Conversely, the SSA, average pore size, and cumulative volume increased to 75 m^2^/g, 63 Å, and 0.08 cm^3^/g, respectively, after calcination at 650 °C (Table S1), possibly due to the removal of CO_2_ trapped in inner closed pores in MW(100) becoming accessible open pores in the MW(650) sample, hence increasing the SSA and the cumulative pore volume. Indeed, the presence of trapped CO_2_ in the MW(100) catalyst and its removal in MW(650) were further confirmed by Fourier transform infrared (FTIR) spectroscopy measurements reported hereafter. Nevertheless, in agreement with PXRD results, the thermal treatment preserved the crystal structure, as demonstrated by the presence of the diffraction fringes in the HR-TEM images collected on the MW(100) and MW(650) samples. Indeed, the spacings among the diffraction fringes, obtained from the measurements of the distances in the Fourier transform (FT) of the images, revealed the presence of the (111), (200), and (220) interplanar spacings of cubic CeO_2_ (JCPDS file number 34-394) in all samples (Figure 1d–f). Depending on the preparation method, different relative abundances of these planes were observed. In particular, as shown in Figure 1d, the MW-assisted preparation leads to the following trend as for the relative abundances: (111) (64%) ≫ (200) (16%) ≫ (220) (3%), whereas the calcination at 650 °C of the same sample (Figure 1e) produced a strong decrease of (111) (33%), which anyway remains the most abundant, accompanied by an increase of both (200) (27%) and (220) (15%, more pronounced). The preparation by conventional heating led to intermediate relative abundances between those obtained for MW(100) and MW(650) (Figure 1f).

Moreover, besides the normal fringes due to the CeO_2_ cubic fluorite-like phase, other phases related to defective ceria have been detected and their relative abundance has also been reported for each sample in Figure 1. More in detail, the analysis of the FT of the images reveals the presence of 15, 24, and 13% of the (101) interplanar spacing of substoichiometric hexagonal Ce_2_O_3_ (JCPDS file number 23-1048) on MW(100), MW(650), and conv(650), respectively. The trend observed indicates that defective ceria particles are formed during preparation with the synthetic procedure, but their relative abundance is promoted by the final calcination at 650 °C of the MW-irradiated material. Interestingly, the (200) interplanar spacing due to the substoichiometric Ce_6_O_11_ monoclinic phase (JCPDS file number 32-196) was detected only in the case of MW(100). This feature can be ascribed to the effect of MW irradiation during the preparation.

### 3.2 Surface Oxygen Vacancy Formation

After having determined the basic structural and textural properties of the three samples, the Ce^3+^ content and catalyst defectivity characterizations are hereafter investigated to discuss the frustrated Lewis pair (FLP) formation.

**Figure 2:**
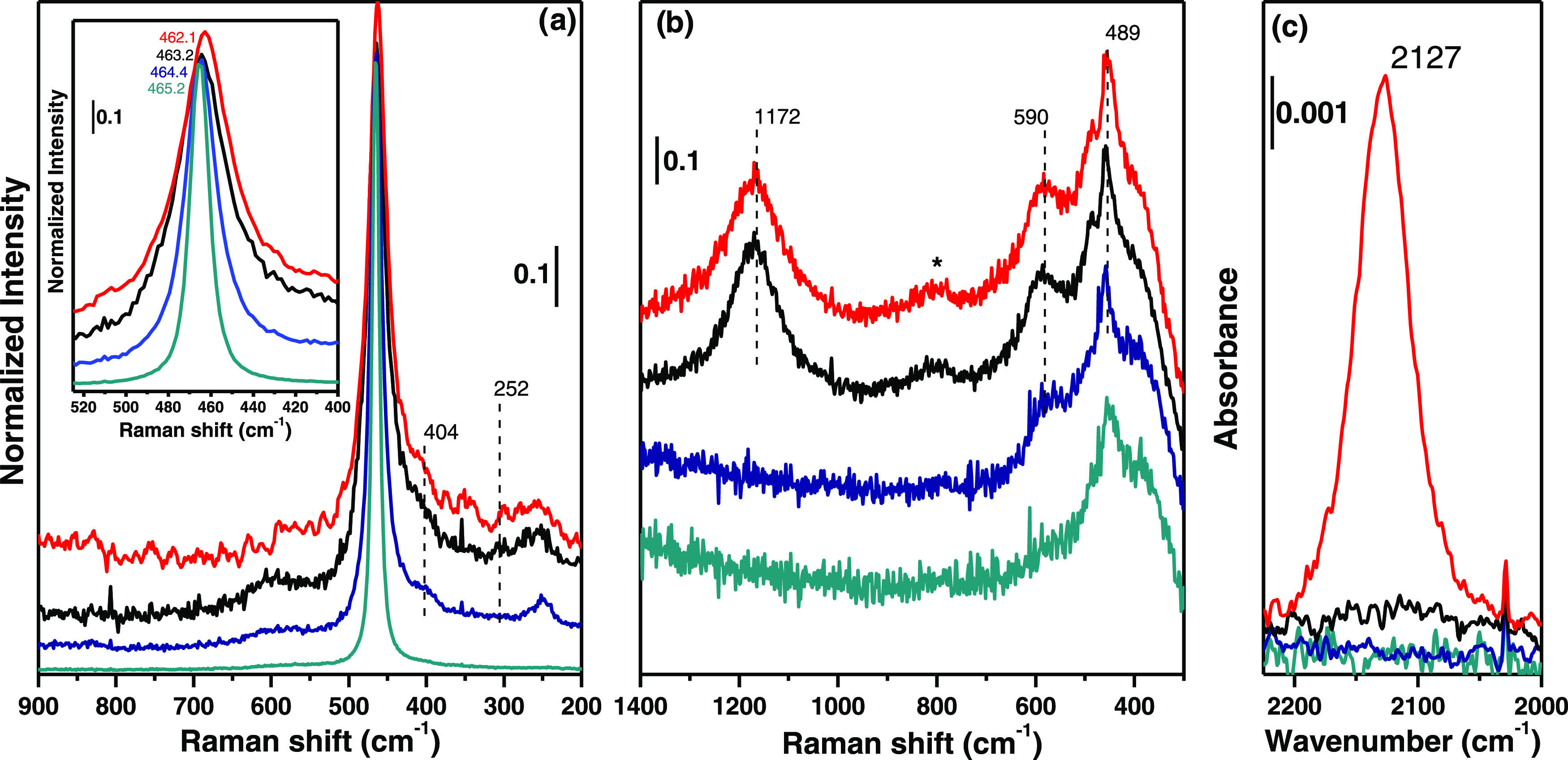
Raman spectra of conv(650) (dark cyan line), MW(650) (dark blue line), MW(100) (black line), and MW(100)-red (red line) measured with (a) 514 nm and (b) 325 nm laser. Details of the CeO_2_ F_2g_ band for the four catalysts and position are reported in the inset of panel (a). Quartz signal from the measurement cell is marked by *. (c) Baseline-corrected Ce^3+ 2^F_5/2_ → ^2^F_7/2_ electronic transition region. FTIR raw spectra are reported in Figure S4.

As described in the SI (Section S2), the FTIR spectra of the four activated samples showed that the activation procedure led to clean CeO_2_ surfaces with isolated hydroxyl groups hereafter discussed for CH_3_OH and CO_2_ adsorption. However, a few important insights into the catalyst’s electronic and textural properties were obtained: (I) the presence/absence of the ν_3_(CO_2_)_as_ band in MW(100) and MW(650), respectively, associated the SSA and cumulative pore volume increase after calcination (Table S1) to the opening of closed pores containing trapped CO_2_;^38^ (II) the Ce^3+ 2^F_5/2_ → ^2^F_7/2_ electronic transition band was observed at 2127 cm^–1^ in MW(100) and MW(100)-red (Figure 2c), indicating the presence of Ce^3+^ in the two samples, being more abundant in the latter than in the former;^39−41^ and (III) a drastic decrease of IR transmitted light after MW(100) reduction, suggesting the formation of V_O_ (Figure S4a), also confirmed by the observation of the Ce^3+^/Ce^4+^ charge transfer at 500–800 nm (CT) in the UV–vis spectrum (Figure S4b).^42,43^

A deeper insight into the catalyst defectivity and the Ce^3+^/Vo presence was obtained through Raman measurements, by exploiting two different laser sources (514 and 325 nm). In particular, the 514 nm source allows a precise identification (position and FWHM) of the F_2g_ mode, while the latter enhances defect signals, being in resonance conditions with such species.^44^ Conv(650) presented a single sharp band at 464 cm^–1^ associated with the Ce–O F_2g_ mode, confirming a not-defective CeO_2_ catalyst (Figure 2a). The full width at half-maximum (FMHM) band increased, and the position of this band downshifted in MW(650) < MW(100) < MW(100)-red, in line with the trends on particle sizes and Ce^3+^/V_O_ concentrations. Since a smaller particle size usually implies the presence of surface Ce^3+^ from coordinative unsaturated sites (CUSs), their straight association with FWHM broadening and F_2g_ shift is not trivial. Following Lee et al.,^45^ we calculated the catalyst (bulk) stoichiometry with eq 1
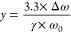
1where the oxygen deficit “*y*” is calculated as a function of the Grüneiser parameter “γ” (γ = 1.24 for CeO_2_) and the band shift “Δω” with respect to the original band frequency “ω_0_” (the frequency value for conv(650) was used).^46^ Considering band position estimation error, the value for the oxygen deficit in MW(650) (*y* = 0.005) can be neglected, associating the F_2g_ broadening and shifting (Table S2) to a direct contribution of smaller particle size and excluding any Ce^3+^ role. On the contrary, MW(100) already presented a considerable amount of Ce^3+^ (as also observed by the IR Ce^3+^ band and further on quantified by XPS; see Table S3), which is reflected by an oxygen deficit value of 0.01. Upon reduction (MW(100)-red), since the same crystallite size is retained (Figure S6) and a similar band broadening is measured (Table S2), the shift of the F_2g_ position could be directly related to the different Ce^3+^ abundance (*y* = 0.02). The F_2g_ band was the only one observed on conv(650); conversely, the microwave-prepared samples, i.e., MW(100/650), presented a wealth of bands, all indicating a local distortion of the ideal cubic CeO_8_ environment (Figure 2a). The bands at 252 and 404 cm^–1^ are related to second-order transverse acoustic vibrations previously associated with the CeO_2_(111) surface longitudinal and transverse Ce–O stretching.^47^ Their intensity increases from MW(100) to MW(650), in line with the higher surface-to-bulk ratio contribution. Parallelly, we observed two more bands at 590 and 1172 cm^–1^ associated with second-order transverse and longitudinal optical transitions, respectively. The *I*_590_/*I*_F_2g__ ratio is more clearly observed in the UV–Raman spectra (Figure 2b) and it is often reported as CeO_2_ defect-meter. However, its quantitative evaluation was not possible due to the convoluted presence of quartz signals from the Raman cell, confirmed by the band at 808 cm^–1^. Nevertheless, it was qualitatively observed that the surface defectivity increases from conv(650) to MW(650), reaching and being the highest for MW(100) samples. Moreover, with resonant-Raman (Figure 2b), we clearly distinguished a band at 489 cm^–1^ in the MW(100) samples. The band was previously associated with Ce^3+^ in the second coordination sphere of an oxygen vacancy,^47^ hence indicating a higher concentration of V_O_ in these samples, in line with the major F_2g_ Ce^3+^-induced shift and Ce^3+ 2^F_5/2_ → ^2^F_7/2_ electronic transition observed in Figure 2a,c, respectively.

**Figure 3:**
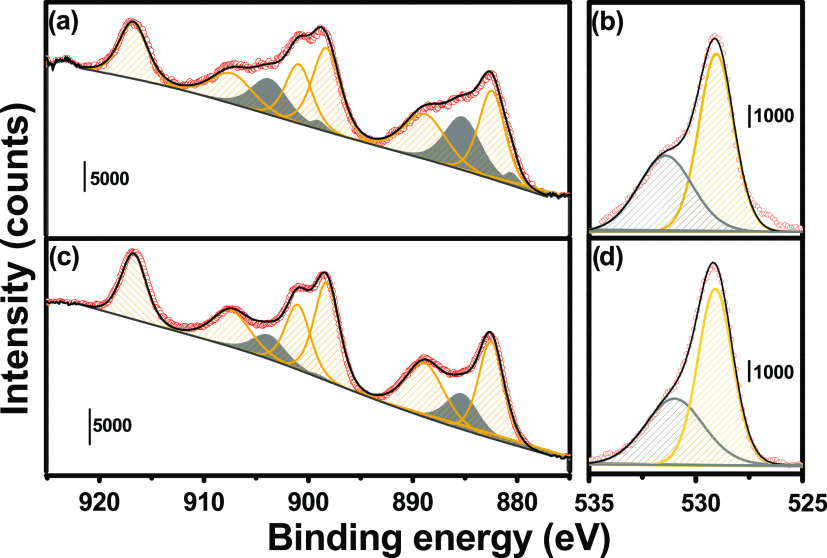
Ex situ (a, c) Ce (3d) and (b, d) O (1s) XPS experimental spectra (red circles) and best fit (black line) of (c, d) MW(100) and (a, b) MW(100)-red. Ce^4+^/O_L_ and Ce^3+^/O^β^ components are indicated by yellow and gray bands, respectively.

To selectively quantify Ce^3+^ formed at the catalyst surface, XPS spectra were collected after CeO_2_ oxidation and reduction at the same temperatures exploited in the previous measurements. Important differences around 885 and 905 eV, where the most intense transitions of Ce^3+^ are located, have been observed in MW(100) and MW(100)-red.^37^ The two spectra, as well as all of the other spectra reported hereafter, were fitted by ten components, six associated with Ce^4+^ transitions and four with Ce^3+^.^37^ After a careful evaluation of Ce^3+^ induced by beam damage (see SI Section 2.1 and Figure S7), we observed that 14% of Ce^3+^ was already present on MW(100) (Figure 3c), in agreement with the presence of the Ce_2_O_3_ phase observed by TEM (Figure 1), while Ce^3+^ increased to 35% after H_2_ treatment (Figure 3a). Even if the Ce^3+^/Ce^4+^ ratio has often been evaluated also from O 1s spectra, other surface species observed by IR spectroscopy might contribute to this spectral region. We then described the O 1s region considering two contributions: a first one at 529.7 eV related to the lattice CeO_2_ oxygen (O_L_) and a second one at a higher energy (≈531 eV), namely, O^β^, potentially originating from a complex convolution of all of the other species, i.e., OH(Ce^4+^), OH(Ce^3+^), CO_3_^=^, and O close to V_O_ (O_Vo_).^48−50^ Since both C 1s (Figure S8) and ex situ IR spectra collected under the same activation conditions did not show an important variation in carbonates and hydroxyl species (the latter actually decrease after reduction, Figure S4b), we can associate the increase of O^β^ in MW(100)-red O 1s spectra (Figure 3b) with a variation of oxygen electronic configuration, i.e., an increase of the surrounding V_O_.

### 3.3 On the Nature and Strength of Ce^3+^–V_O_ Sites

**Figure 4:**
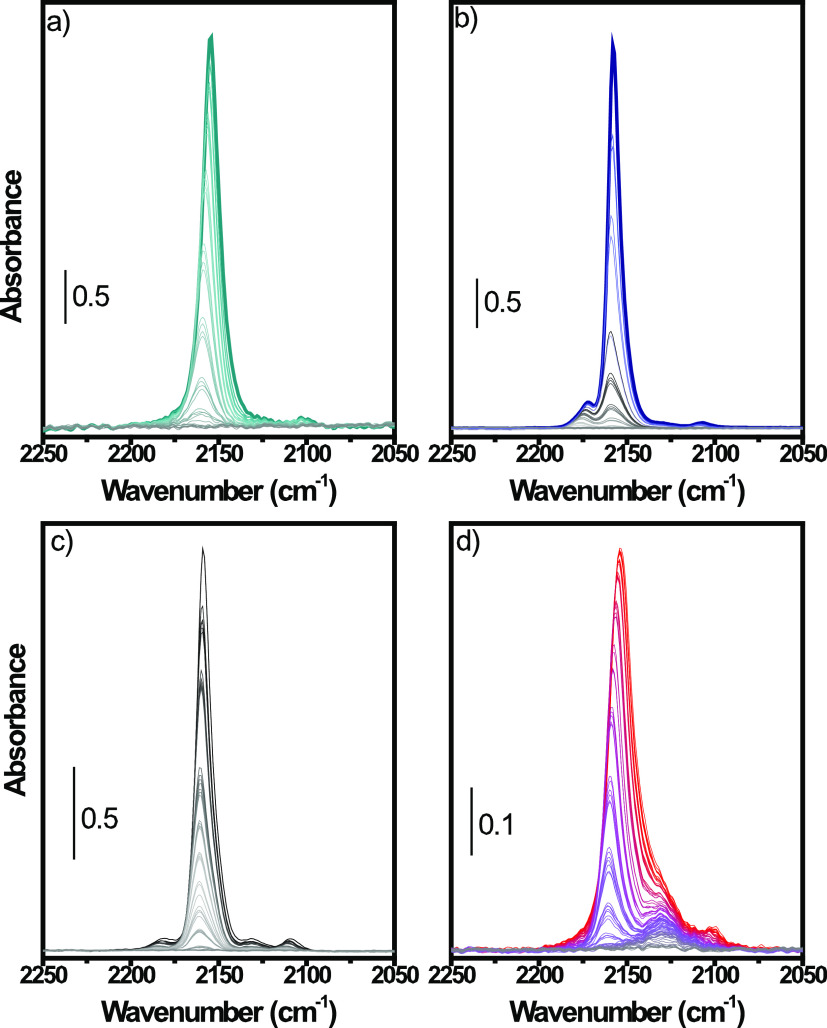
Difference IR spectra of CO increasing partial pressure at LNT over (a) conv(650), (b) MW(650), (c) MW(100), and (d) MW(100)-red. The CO partial pressure increases from the gray to the colored line. The spectrum of the material prior to interaction with CO has been subtracted. For the sake of brevity, the IR band assignment has been omitted in the main text and is reported in the SI.

After having exploited both bulk and surface-sensitive techniques to probe Ce^3+^ and defect sites, CO chemisorption at LNT (nominal temperature of −193 °C) and monitored by IR spectroscopy was employed to investigate the first surface-layer oxidation state and geometry and to qualitatively evaluate surface Ce^3+^ abundance. As described in SI Section 2.2.1, the CO stretching vibration is influenced by the electron-donor/-acceptor behavior of the surface. First of all, CO interacting with hydroxyl groups was not observed in any of the catalysts, as shown by the unaltered ν(OH) band after CO adsorption (Figure S9). This implies that the OH might have a basic character and/or are not accessible to the CO probe. IR spectra collected at increasing CO partial pressure at LNT over conv(650) (Figure 4a) showed a single band at 2154 cm^–1^, indicating the presence of nondefective Ce^4+^ sites and a weak band at 2101 cm^–1^ associated with ^13^CO contribution since ν(^13^CO–Ce^+4^)/ν(^12^CO–Ce^+4^) ≈ 0.976.^51^ Moving to MW(650) (Figure 4b), an extra band at higher wavenumbers (≈2171 cm^–1^) is observed, related to Ce^4+^ coordinative unsaturated sites (CUSs), i.e., kink and edge sites.^39,52,53^ CO adsorption over MW(100) (Figure 4c) shows (I) a band at 2181 cm^–1^ related to CUS sites, blue-shifted with respect to MW(650), suggesting sites with a higher degree of unsaturation/stronger Lewis acidity; (II) a main band at 2159 cm^–1^ related to platelike Ce^4+^ sites (and its satellite ^13^CO contribution at 2108 cm^–1^); and (III) a weaker band at 2131 cm^–1^ assigned to CO–Ce^3+^, in agreement with its back-donating character and literature results.^39,53^ Eventually, MW(100)-red gives rise to a different situation (Figure 4d), not only in terms of spectra components but also in terms of their intensities (spectra were collected on the same pellet, so intensities are quantitatively comparable). In particular, the spectra obtained on the reduced sample undergo a substantial decrease in intensity in the full mid-IR range (Figure S5a). Moreover, CO predominantly interacted with less defective Ce^4+^ (2154 cm^–1^), with a minor contribution of more defective sites (2177 cm^–1^), and the Ce^3+^/Ce^4+^ intensity ratio increased by three times (Figure S10), highlighting the higher abundance of reduced metal sites. Due to their consumption, Ce^4+^ CUS sites originating the 2181 cm^–1^ band in MW(100) then act as precursors for the reduction of Ce^4+^ to Ce^3+^ at 150 °C in H_2_. Moreover, CO interaction with the MW(100)-red Ce^3+^ sites occurs at a lower coverage with respect to MW(100), suggesting a stronger interaction in the former compared to the latter.

### 3.4 CH_3_OH and CO_2_ Activation over Ce^3+^/V_O_ and FLP

**Figure 5:**
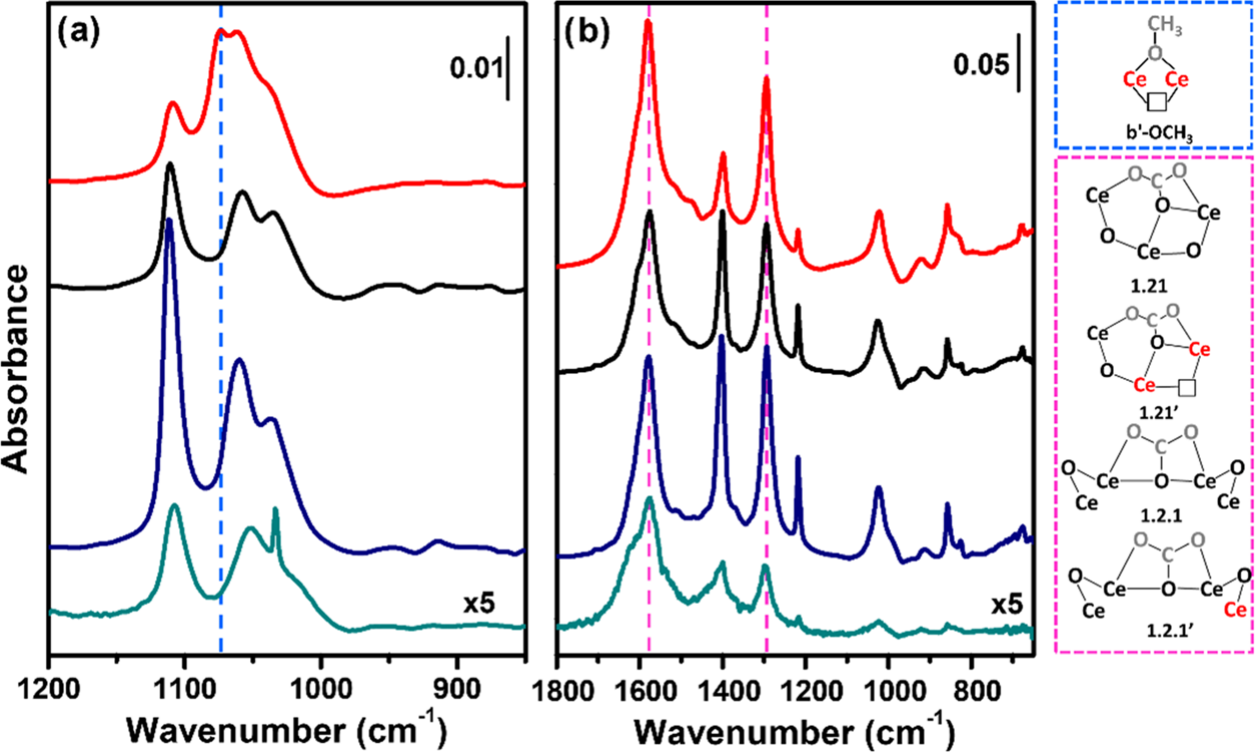
Difference IR spectra of (a) 3 mbar of methanol and (b) 100 mbar CO_2_ RT adsorption over conv(650) (dark cyan line), MW(650) (dark blue line), MW(100) (black line), and MW(100)-red (red line) catalysts. The spectrum of the material prior to interaction with CH_3_OH/CO_2_ has been subtracted. Sketched methoxide and carbonate species formed over CeO_2_ are shown in blue and purple panels, respectively. CH_3_OH and CO_2_ atoms are shown in gray, Ce^3+^ in red, and oxygen vacancy with black squares. The carbonate nomenclature, based on the number of cerium ions binding each carbonate oxygen atom, was taken from Vayssilov et al.;^56^ the apostrophe is here used to indicate carbonates considering Ce^3+^ presence. Full-range IR spectra are reported in the SI for the sake of clarity in Figures S12 and S14.

The evaluated surface and bulk Ce^3+^ content and the defectivity confirmed that MW(100)-red presented sufficient Ce^3+^/V_O_ sites on its surface to hypothesize the formation of FLP. However, Ce^3+^/V_O_ clusters should be clustered over CeO_2_ (110) planes to affect small-molecule activation, e.g., CO_2_ adsorption as a reactive carbonate.^9,14^ Methanol and carbon dioxide absorption was then studied by FTIR to corroborate this point. Since their adsorption over CeO_2_ catalysts is well documented in the literature, the band assignment is thoughtfully described in SI Sections 2.2.2 and 2.2.3. After methanol adsorption (Figure 5a) over the CeO_2_ samples, besides the formation of the usual methoxide species (terminal, bibridged, and tribridged, described in SI section 2.2.2), we observed a band located at 1073 cm^–1^ over the MW(100)-red sample (Figure 5a red line), associated with a methoxide group bridging two Ce^3+^ atoms without interacting with the V_O_ (*b*′-OCH_3_), as sketched in Figure 5 (blue panel). Indeed, the CeO_2_ reduction process is well-known to cause a blue-shift of *b*-OCH_3_ ν(CO) due to different charge delocalizations over the methoxide oxygen atom, i.e., Ce^3+^ polarizes and delocalizes less than Ce^4+^, causing an increase of the C–O bond order and shifting ν(CO) to higher energies.^54^ Moreover, MW(100)-red presented a lower *m*-OCH_3_/*b*-OCH_3_ intensity ratio than the ideal one (2:1) as occurs for the other catalysts, unveiling a preferential reduction of (100) and (110) faces, where *b*-OCH_3_ are more stable. The preferential reduction of (110) planes, coupled with the observed CO–Ce^3+^ strong interaction (see Section 3.3) and the high Ce^3+^ concentration (>30%), confirmed Ce^3+^–V_O_ clustering over the desired plane to form frustrated Lewis pairs (FLPs).^55^ It is noteworthy that the Ce^3+^ electronic transition at 2127 cm^–1^ was not modified by methanol adsorption (Figure 7c), suggesting, as confirmed below by XPS (vide infra), that *b*′-OCH_3_ formation did not modify the cerium oxidation state.

Parallelly, after CO_2_ and ^13^CO_2_ adsorption (carefully described in SI Section 2.2.3), on the basis of a direct comparison with the most recent literature,^56^ we restricted the formed carbonates to four bidentate species (named after Vayssilov et al.^56^) sketched in Figure 5 for clarity (purple panel). Moreover, on MW(100)-red catalysts, we observed that CO_2_ adsorption caused a (I) consumption of Ce^3+^ electronic transition at 2127 cm^–1^, suggesting a Ce^3+^/CO_2_ electronic interaction (Figures 7a, S14d and S15b); and (II) a higher carbonate-to-bicarbonate ratio with respect to the other catalysts (Figure 5b), indicating, as rationalized hereafter, an increase of the 1.21′ carbonate associated with the higher Ce^3+^/V_O_ content.

The Ce^3+^/CO_2_ electronic interaction was further confirmed by comparing UV–Vis (Figure S17a) and XPS spectra of MW(100)-red before (purple line) and after (dark red line) interaction with CO_2_ (Figure 7a,c). Both measurements showed a decrease of (I) Ce^3+^/Ce^4+^ CT (Figure S17a) and (II) Ce^3+^(3d) peaks after CO_2_ adsorption at RT and 30/150 °C, respectively. Moreover, the CO signal detected during CO_2_ interaction in the latter measurement (Figure S20) did not show any significant variation. In addition, O 1s XPS peaks (Figure S17b) showed that O_L_ and O^β^ signals did not undergo a considerable variation after CO_2_ adsorption, suggesting that the latter did not modify the O electronic reconfiguration that occurred after reduction (vide supra), meaning that the formed carbonate did not fill the V_O_, as instead previously hypothesized.^24^

**Figure 6:**
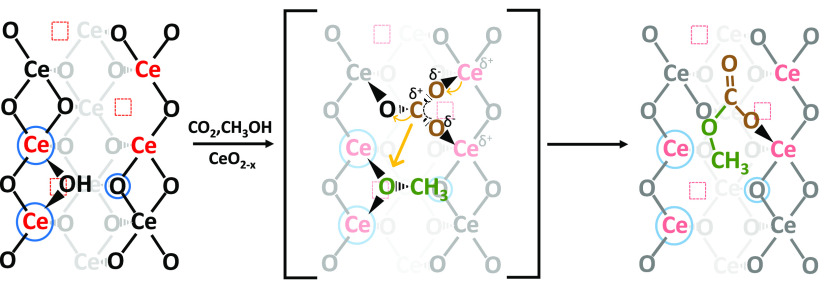
Sketched CO_2_ (brown) and CH_3_OH (green) reaction over the CeO_2_ surface with Ce^4+^ (black), Ce^3+^ (red), V_O_ (red squares), and FLP (blue circle) to form MMC.

Through these observations, it is deduced that the Ce^3+^/CO_2_ electronic interaction must then occur through the formation of a negatively charged carbonate, allowing Ce^3+^-to-CO_2_ charge redistribution, preventing V_O_ occupation (Figure 6). Even if by IR measurements it was not possible to isolate a single carbonate between the four reported in Figure 5, coupling IR with UV–Vis and XPS results, we observed that the b-CO_3_^=^/hCO_3_^–^ ratio increased parallel to the Ce^3+^ content in the order MW(100)-red > MW(100) > MW(650) > conv (650). Considering then that (I) only one of the four carbonates identified in Figure 5 implied CO_3_^=^ formed over Ce^3+^/V_O_; (II) bidentate carbonate abundance increased with the Ce^3+^ content; (III) Ce^3+^ fingerprints, i.e., the IR 2127 cm^–1^ band, Ce^3+^/Ce^4+^ UV–Vis CT, and Ce^3+^(3d) peaks have been consumed after CO_2_ adsorption; and (IV) V_O_/CO_2_ interaction was not observed by O 1s spectra, we can hypothesize that the carbonate 1.21′ concentration increased with the Ce^3+^ content reaching the highest concentration over MW(100)-red. Indeed, this bidentate carbonate would allow Ce^3+^-to-CO_2_ charge redistribution without filling the oxygen vacancy. Moreover, it is noteworthy that this carbonate species, hypothesized by combining the experimental IR data of this work with theoretical results reported in the literature, perfectly matches that expected in the case of CO_2_ adsorbed over FLP sites (Figure 6).^14,56^

**Figure 7:**
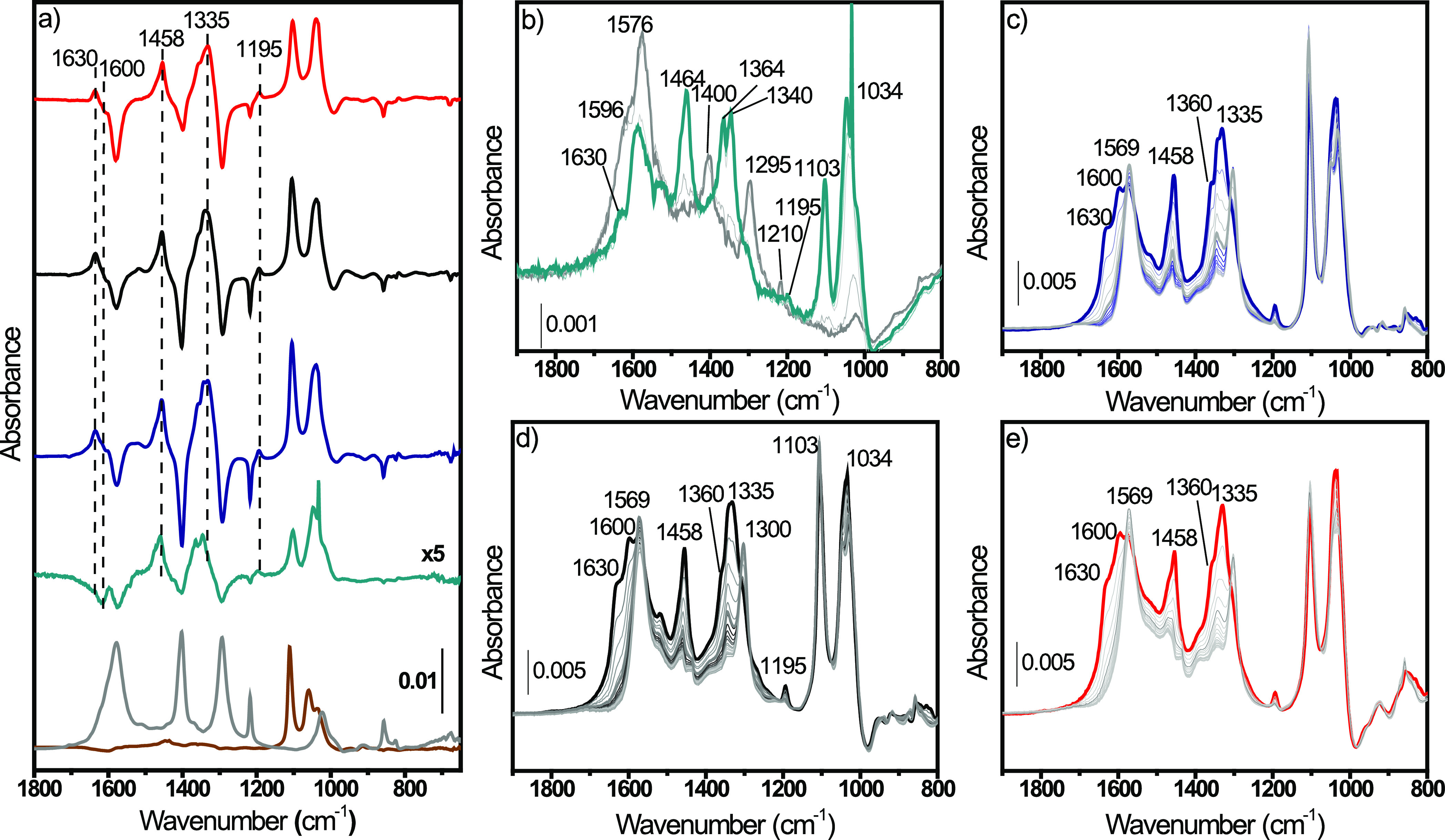
(a) Difference IR spectra of conv(650) (dark cyan line), MW(650) (dark blue line), MW(100) (black line), and MW(100)-red (red line). Spectra of each CO_3_–CeO_2_ component have been subtracted. CH_3_O-MW(650) (gray line) and CO_3_-MW(650) (brown line) components are shown for clarity. (b–e) FTIR spectra evolution of adsorption of methanol (3 mbar) over (b) conv(650), (c) MW(650), (d) MW(100), and (e) MW(100)-red previously exposed to 100 mbar of CO_2_. Adsorption time evolution goes from the gray line to the colored line. The full range of the as-measured spectra is reported in Figure S18.

To verify the effective CO_2_ and CH_3_OH activation over FLP, we exploited their reactivity to form monomethylcarbonate (MMC), by studying the CO_2_/CH_3_OH chemical interaction with the CeO_2_ surface previously saturated with methoxide (CH_3_O–CeO_2_) or carbonate (CO_3_–CeO_2_) species, respectively. The presence of Ce^3+^ alone should indeed hamper MMC formation,^29,30^ while clustered Ce^3+^/V_O_ forming an FLP is expected to improve the CO_2_ reactivity.^32^ A preliminary comparison between CO_3_-MW(650) and CH_3_O^–^MW(650) (taken for simplicity as reference spectra) and the spectral differences of CO_3_–CeO_2_ after CH_3_OH adsorption (Figures 7a and S19a) indicated a consumption of b-CO_3_^=^ and hCO_3_^–^ species and formation of methoxide vibrations. The spectra time evolution (Figures 7b–e and S19b–e) confirmed that the intensity of CO_3_^=^ and hCO_3_^–^ bands located at 1300, 1364, and 1576 cm^–1^ decreased. Carbonate consumption was followed by the formation of *m*-OCH_3_ and *t*-OCH_3_ modes at 1034 and 1103 cm^–1^ (Figures 7b–e and S20) and at 2804 and 2812 cm^–1^ (Figure S19 b–e), respectively (see Table S5 for a detailed band assignment). The former intensity rapidly decreased, while the latter increased. Besides the *m*-OCH_3_ → *t*-OCH_3_ interchange, we observed the appearance of different bands at around 1195, 1333, 1454, 1600 cm^–1^ (Figures 7b–e and S20) and 2887, 2956 cm^–1^ (Figure S19 b–e), indicating the formation of MMC (see Table S5 for a detailed band assignment). The kinetic evolution of the bands indicates that *m*-OCH_3_ reacts with CO_3_^=^ species to form MMC, while Ce^4+^–O sites available after *m*-OCH_3_ consumption are covered by *t*-OCH_3_ species.^19^ In parallel to MMC band formation, we observed the growth of three bands at around 1360, 1630, and 2942 cm^–1^ related to formate (HCOO^–^).^56^ The appearance of formate species can be related to methanol dehydrogenation and to the parallel ceria reduction.^57−61^ It is noteworthy that both HCOO^–^ and MMC bands’ formation kinetics (Figures 7b–e and S10 b–e) and their final intensities (Figures 7a and S19a) were very similar for all of the samples. As will be clarified hereafter, the absence of differences between the samples is associated with the Ce^3+^ content, which was reduced through 1.21′ carbonate formation.

**Figure 8:**
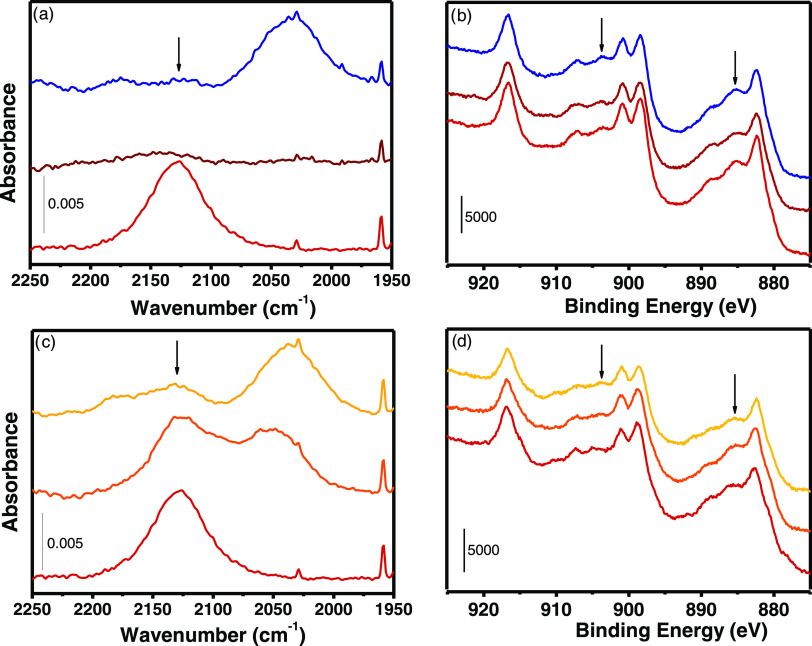
Ex situ (a, c) Ce^3+^ FTIR ^2^F_5/2_ → ^2^F_7/2_ band and (b, d) Ce 3d XPS experimental spectra of the MW(100)-red catalyst (red line) after interaction with (a, b) CO_2_ (dark red line) followed by CH_3_OH (blue line) and (c, d) CH_3_OH (orange line), followed by CO_2_ (yellow line). Most Ce^3+^ fingerprints are indicated with arrows.

To monitor Ce^3+^–V_O_ evolution during CO_2_ and CH_3_OH adsorption sequence, XPS spectra were collected at 30 °C (Figure 8b,d). Ce^3+^ peaks decreased after CO_2_ adsorption (Figure 8b dark red line) and increased upon interaction with CH_3_OH (Figure 8b blue line). While the former was described above and associated with Ce^3+^ partial reoxidation due to 1.21′ carbonate formation, the latter can be associated either with Ce^4+^ reduction caused by methanol-to-formate oxidation or with MW(100) beam damage (see SI Section 2.1).^59−61^ Since both effects were observed, it is difficult to ascribe Ce reduction to one of them.

**Figure 9:**
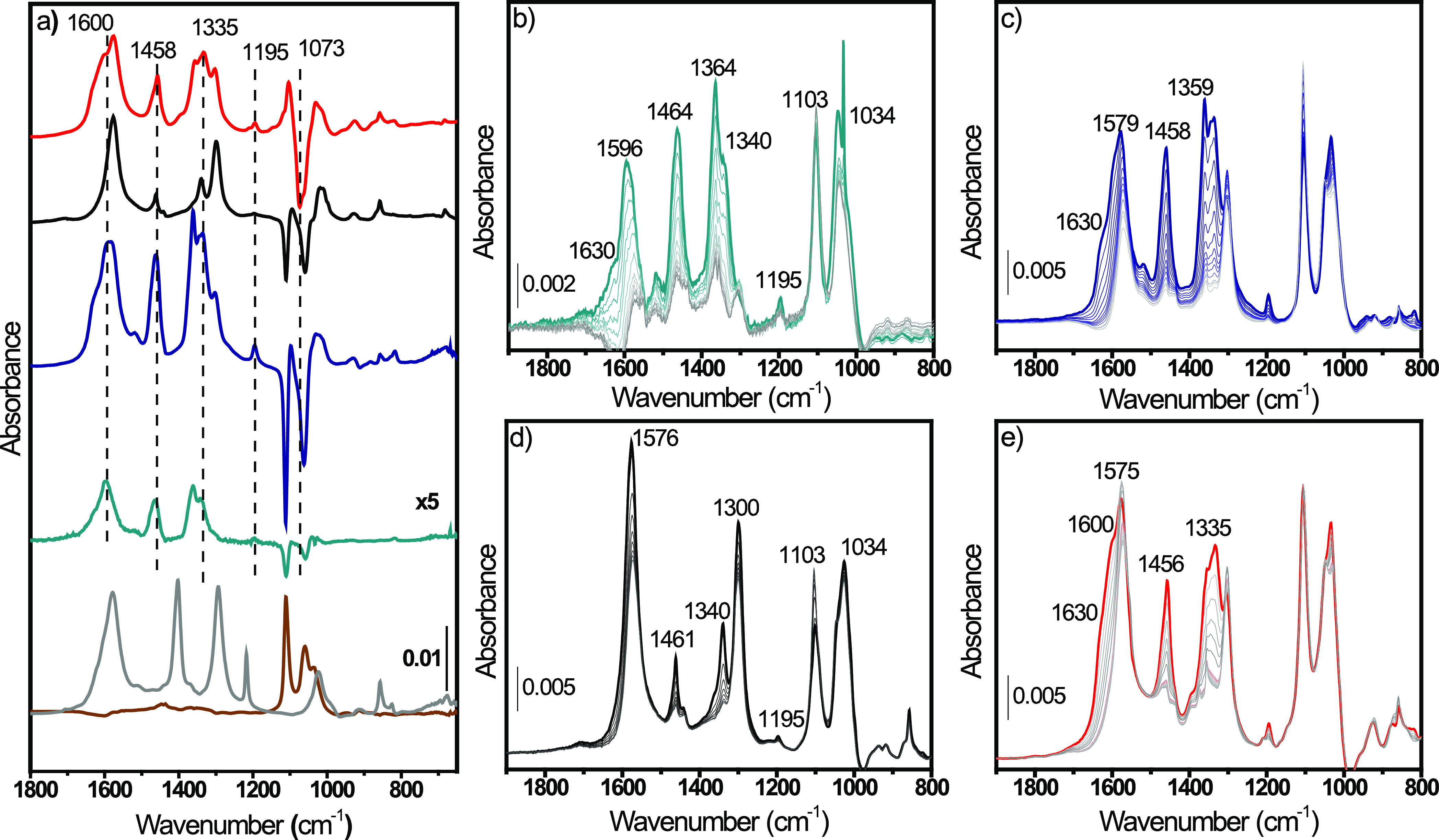
(a) Difference IR spectra of conv(650) (dark cyan line), MW(650) (dark blue line), MW(100) (black line), and MW(100)-red (red line). Spectra of each CH_3_O–CeO_2_ component have been subtracted. CH_3_O-MW(650) (gray line) and CO_3_-MW(650) (brown line) components are shown for clarity. (b–e) FTIR spectra evolution of adsorption of CO_2_(100 mbar) over (b) conv(650), (c) MW(650), (d) MW(100), and (e) MW(100)-red previously exposed to 3 mbar of CH_3_OH. Adsorption time evolution goes from the gray line to the colored line. The full range of the as-measured spectra is reported in Figure S21.

While the formed carbonates did not show major changes in the reactivity with methanol, the reverse interaction, i.e., CO_2_ interacting with a surface rich in methoxide species, showed a different behavior. As for the previous case, a qualitative analysis of the difference spectra obtained upon CO_2_ adsorption over CH_3_O–CeO_2_ samples (Figures 9a and S22a) indicated consumption of methoxide species (1103, 1034, 2804, and 2812 cm^–1^) and parallel formation of carbonates (1300, 1364, and 1576 cm^–1^), MMC (1195, 1333, 1454, 1600, 2887, and 2956 cm^–1^), and formates (1360, 1630, and 2942 cm^–1^) (see Table S5 for the detailed band assignment).^19,55,56^ The spectra evolution of the four samples (Figures 9b–e and S22b–e) indicated a rapid formation of carbonates, in line with their quick adsorption kinetic.^19^ Carbonate adsorption is however limited by the slower rise of MMC and formate vibrations parallel to the consumption of *m*-OCH_3_. While in the previous scenario (i.e., CO_2_ adsorbed over CH_3_O–CeO_2_) relevant differences were not observed, in this case, the spectra difference (Figures 9a and S22a) and their kinetic evolution (Figures 9b–e and S22b–e) indicated major variations coupled with the Ce^3+^ content. First, conv(650) and MW(650) were very similar, indicating that the different surface defectivity did not affect the MMC formation rate. Contrarily, on MW(100) and MW(100)-red, the spectra evolution presented mostly carbonates in the former and more MMC in the latter. The differences in the MMC formation are clearly observable from the difference spectra where the CH_3_O–CeO_2_ spectrum was subtracted (Figures 9a and S22a). We clearly observed that MMC was almost absent in MW(100), confirming that the low Ce^3+^ content (≈14%) poisoned the reaction.^29,30^ Following these results, MW(100)-red with Ce^3+^ >30% should present an even lower intensity of MMC. Conversely, we observed a qualitatively higher concentration of MMC (Figure 9a) together with the consumption of *b*′-OCH_3_ species (1073 and 2789 cm^–1^) and a decrease of the Ce^3+ 2^F_5/2_ → ^2^F_7/2_ electronic transition (Figures 8c and S22a). These indirectly suggested a Ce^3+^ oxidation upon CO_2_ adsorption at CH_3_O-MW(100)-red. Additionally, Ce(3d) XPS spectra (Figure 8d) showed (I) a partial increase of Ce^3+^ bands after CH_3_OH adsorption (orange line) and (II) a Ce^3+^ partial consumption after subsequent CO_2_ adsorption (blue line). These results confirmed that Ce^3+^/CO_2_ interaction occurred even on a methoxide-rich CeO_2_ surface since formation of *b*′-OCH_3_ did not oxidize Ce^3+^ species. Additionally, the agreement between XPS and Ce^3+ 2^F_5/2_ → ^2^F_7/2_ IR transition confirmed that the latter could be used to qualitatively monitor the cerium oxidation state during reactions as we recently showed.^62^ FLP allowed then the parallel presence of polarized 1.21′ carbonates and *b*′-OCH_3_. The former possesses a more electrophilic carbon, while the latter is characterized by a highly polarized oxygen, overall resulting in an improved MMC formation (Figure 6). On the other hand, no differences were observed in MMC formation when CO_2_ was adsorbed first (Figure 7) since Ce^3+^ oxidation prevented further *b*′-OCH_3_ formation, hence hindering any reactivity differences. Notably, since methanol dehydrogenation occurs on Ce^4+^ sites, while the reverse reaction takes place on Ce^3+^, the presence of formate vibrational modes depends on the Ce^3+^ content. In line with this, formate species were observed on conv(650) and MW(650), whereas they were not detected on MW(100). In contrast, MW(100)-red presented formate vibrational modes, suggesting that the FLP site might have an active role in methanol dehydrogenation as well.

## 4 Conclusions

In conclusion, CO_2_ conversion to MMC was investigated over four CeO_2_ samples with different defects and Ce^3+^ contents. The defectivity was observed to increase as conv(650) < MW(650) < MW(100) ≈ MW(100)-red, while Ce^3+^ increased as conv(650) ≈ MW(650) < MW(100) < MW(100)-red. The four samples presented different SSAs, which affected their spectroscopic transparency, however, without presenting a correlation with CO_2_ and CH_3_OH activations. Contrarily, the presence of defects without Ce^3+^ (MW650) improved the surface reactivity toward MMC with respect to a sample not presenting defects (conv(650)). The parallel presence of defects and Ce^3+^ <30% (MW(100)) hindered MMC formation, indicating that isolated Ce^3+^ sites do not favor the reaction. On the contrary, in the case of defects and Ce^3+^ >30% (MW(100)-red), MMC formation was higher than in MW(100) and associated with the presence of frustrated Lewis pairs. FLP formation was logically concluded after having observed (I) Ce^3+^ ≈ 35%, (II) Ce^3+^–V_O_-rich (110) planes, (III) formation of *b*′-OCH_3_ after CH_3_OH adsorption, and (IV) abundant 1.21′ carbonate upon CO_2_ adsorption. Moreover, Ce^3+^ consumption following CO_2_/FLP interaction indicated the formation of a polarized 1.21′ carbonate where the carbon atom is more electrophilic. Contrarily, CH_3_OH/FLP interaction occurs without Ce^3+^ consumption through the formation of *b*′-OCH_3_, where the oxygen atom is more nucleophilic. A mechanism describing 1.21′ carbonate and *b*′-OCH_3_ parallel formation and interaction is proposed. Additionally, a possible role of FLP in methanol dehydrogenation to formate species was suggested. The involvement of FLP in the formation of formates will be further investigated in the future. Lastly, we reported that the Ce^3+^ IR band occurring at 2127 cm^–1^ can be easily used to track the Ce oxidation state.

## Supporting Information Available

All of the raw IR and XPS spectra (PDF)
