# Supplementary material for: CeO2 Frustrated Lewis Pairs Improving CO2 and CH3OH Conversion to Monomethylcarbonate
Source: ACS Appl Mater Interfaces. 2023 Mar 14;15(12):15396–408. doi: 10.1021/acsami.2c22122 (PMC10064321; doi:10.1021/acsami.2c22122)
Supplement: Supplementary file 1 — am2c22122_si_001.pdf [file am2c22122_si_001.pdf]

Supporting Information

CeO_2 Frustrated Lewis Pair improving CO_2 and CH_3OH conversion to Monomethylcarbonate

Davide Salusso[1,2], Giorgio Grillo[3], Maela Manzoli[3], Matteo Signorile[1], Spyridon Zafeiratos[4], Mathias Barreau[4], Alessandro Damin[1], Valentina Crocellà[1], Giancarlo Cravotto[3] and Silvia Bordiga[1]*

[1]Department of Chemistry, NIS Center and INSTM Reference Center, University of Turin, 10125, Turin, Italy.

[2] European Synchrotron Radiation Facility, CS 40220, 38043 Grenoble Cedex 9, France

[3]Department of Drug Science and Technology, University of Turin, Turin 10125, Italy

[4]Institut de Chimie et Procédés pour L'Energie, L'Environnement et La Santé, UMR 7515 CNRS-UdS, 25 Rue Becquerel, 67087 Strasbourg, France

*Email : silvia.bordiga@unito.it

Table of contents

CeO_2 Frustrated Lewis Pairs improving CO_2 and CH_3OH conversion to Monomethilcarbonate 1
1 Textural, structural and morphological properties of the catalysts 2
2 Structural and spectroscopic characterizations 4
2.1 XPS beam damage evaluation and spectra fitting results 6
2.2 CO, CO_2 and CH_3OH adsorption investigated by FTIR 8
2.2.1 CO adsorption 8
2.2.2 CH_3OH adsorption 10
2.2.3 CO_2 adsorption 11
2.2.4 CH_3OH adsorption over CeO_2 previously saturated with CO_2 (CO_3-CeO_2) 15
2.2.5 CO_2 adsorption over CeO_2 previously saturated with CH_3OH (CH_3O-CeO_2) 17

…………………

1 Textural, structural and morphological properties of the catalysts

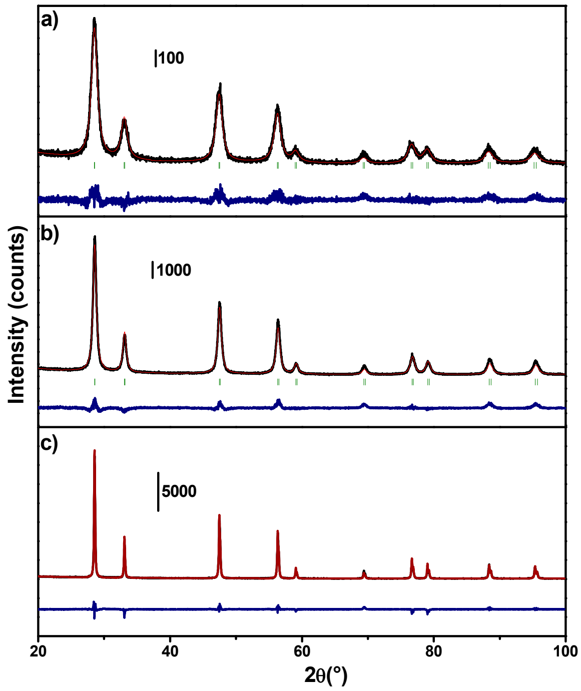

Figure S1. PXRD experimental (black line), refined (red line) patterns and difference curve (blue line) of a) MW(100), b) MW(650) and c) conv(650).

Table S1. Structural and Surface area properties for conv(650), MW(100) and MW(650).

	Conv(650)	*MW(100)*	*MW(650)*
Space group	Fm-3m	Fm-3m	Fm-3m
Refined Crystallite size (nm)[TEM]	61 [10]	6 [4.1±1.2]	10 [8.1±2.2]
BET Specific Surface Area (m^2/g)	8	43	75
Average Pore Size (Å)	47	50	63
Cumulative Pore Volume at 100 Å (cm^3/g)	<0.01	0.05	0.08

Table S2. Details of CeO_2-X catalysts Raman Spectra collected with 514 nm laser.

	Conv(650)	*MW(650)*	*MW(100)*	*MW(100)-red*
F_{2g} position(cm^{-1})	465.22 ± 0.05	464.49 ± 0.10	463.6 ± 0.3	462.30 ± 0.13
F_{2g} FWHM(cm^{-1})	10.43 ± 0.16	16.8 ± 0.4	26 ± 1	26.3 ±0.5
Oxygen deficit (y)	0	0.005	0.01	0.02
CeO_{2-y}	CeO_2	$CeO_{1.995}$	$CeO_{1.99}$	$CeO_{1.98}$

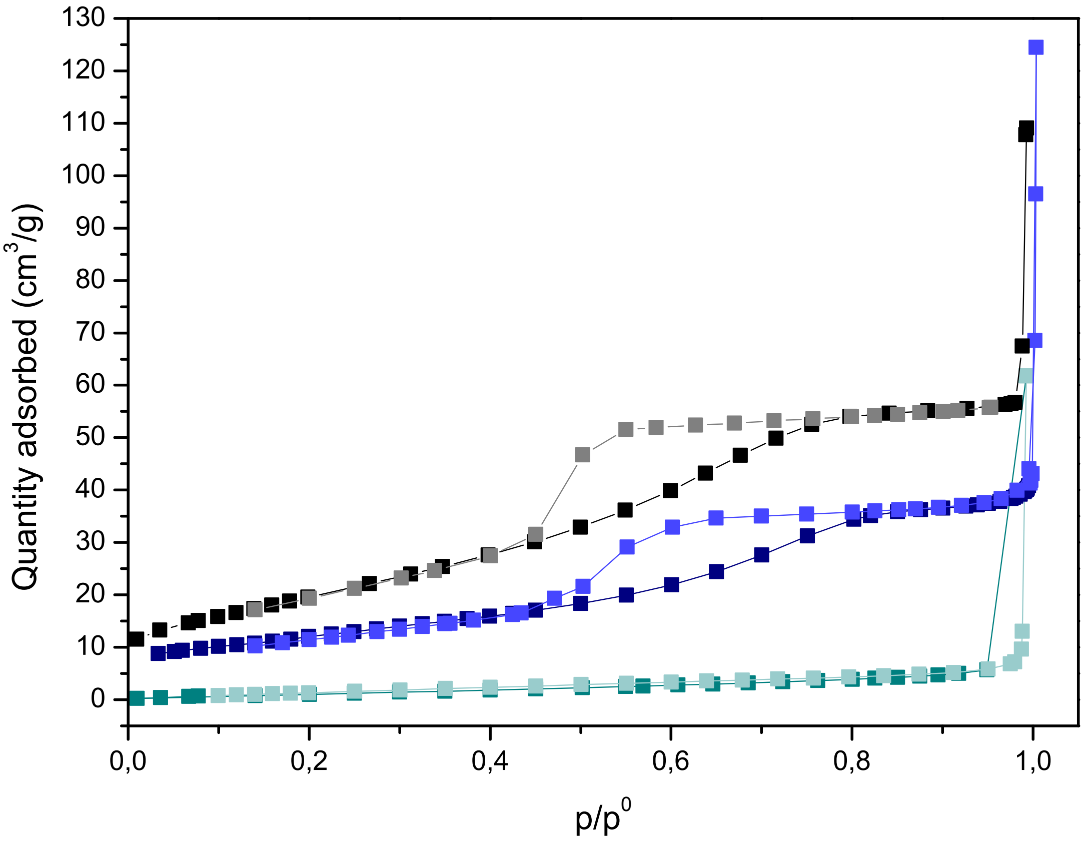

Figure S2. N_2 adsorption/desorption isotherms in darker and lighter colours, respectively, for conv(650) (light blue), MW(650) (black) and MW(100) (blue).

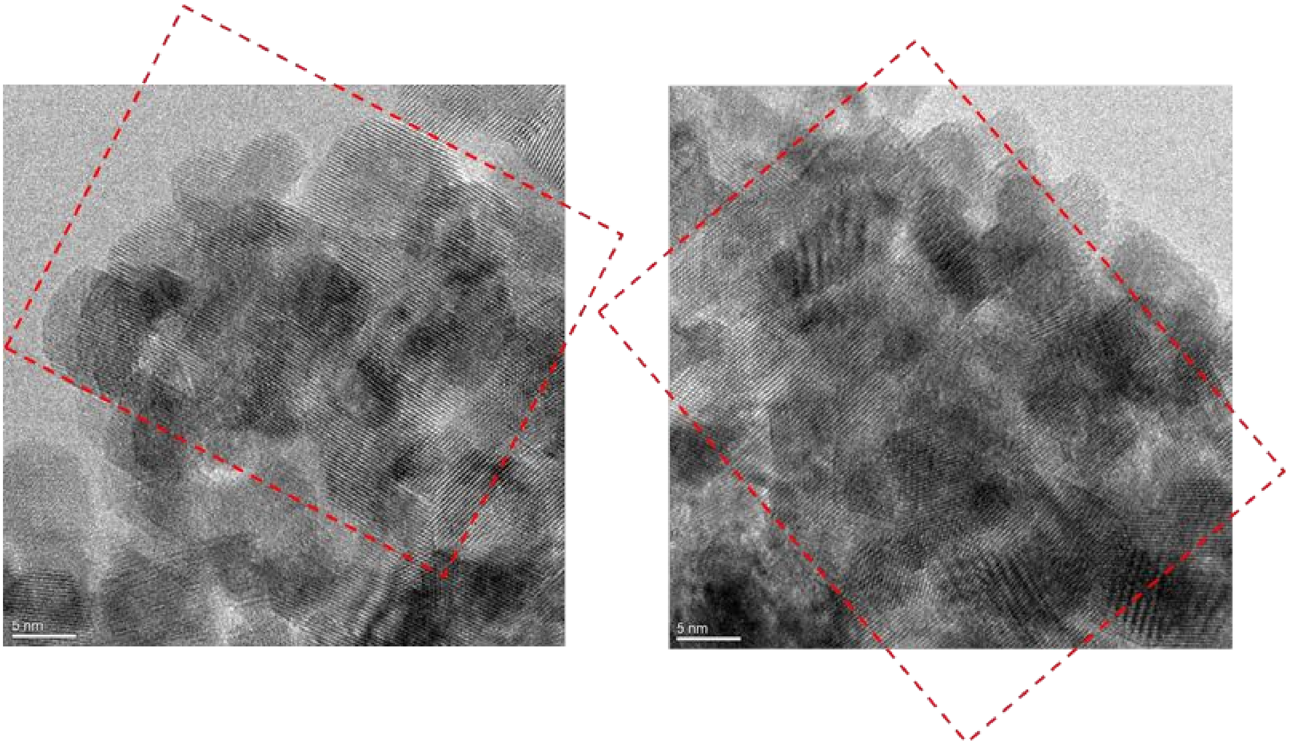

Figure S3. HR-TEM images of CeO_2-conv. Instrumental magnification: 500000×.

The SSA of the conv(650) sample is 8 m^2/g, but the sample appears crystalline and made up by nanoparticles with size of around 10 nm. However, a closer inspection reveals the presence of extended regions with the same orientation of the diffraction fringes (highlighted by the red dashed boxes in Figure 3): this feature indicates that upon conventional synthesis, i.e. calcination at 650 °C for 8 hours, strong agglomeration of the nanoparticles occurred, which resulted in a strong decrease of the SSA, in line with PXRD refinement showing an average crystallite size of 61 nm (Table S1).

2 Structural and spectroscopic characterizations

The IR spectrum of conv(650) material, measured after O_2 activation, presents a peculiar profile i.e., flat scattering profile parallel to a reverse band around 1000 cm^{-1}, in line with its low SSA making it less transparent to IR radiation, and few features that can be ascribed to some organic fragments present on the surfaces (dark cyan line in Figure S4a). As detailed in the experimental section 2.2, samples undergo O_2/H_2 activation procedures to remove organic residuals and to obtain a better-defined structure. A band at 2342 cm^{-1}, observed in MW(100) catalysts was assigned to $\nu_3(CO_2)_{as}$ indicating the presence of CO_2 produced during the synthesis (from urea decomposition) and trapped in the catalysts crystallites.[1] The absence of the band in MW(650) indicates that the calcination process removes trapped CO_2 increasing its catalyst SSA and cumulative pore volume (Table S1). Part b) of Figure S4 illustrates the enlarged view of the ν(O-H) region. Whilst all the samples presented isolated terminal, bi-bridged and tri-bridged hydroxyls groups, identified from ν(O-H) at

3710, 3685 and 3657 cm^{-1}, respectively, the former was not observed on MW(100)-red (Figure S4 red line), consequence of CeO_2 surface partial reduction.[2,3]

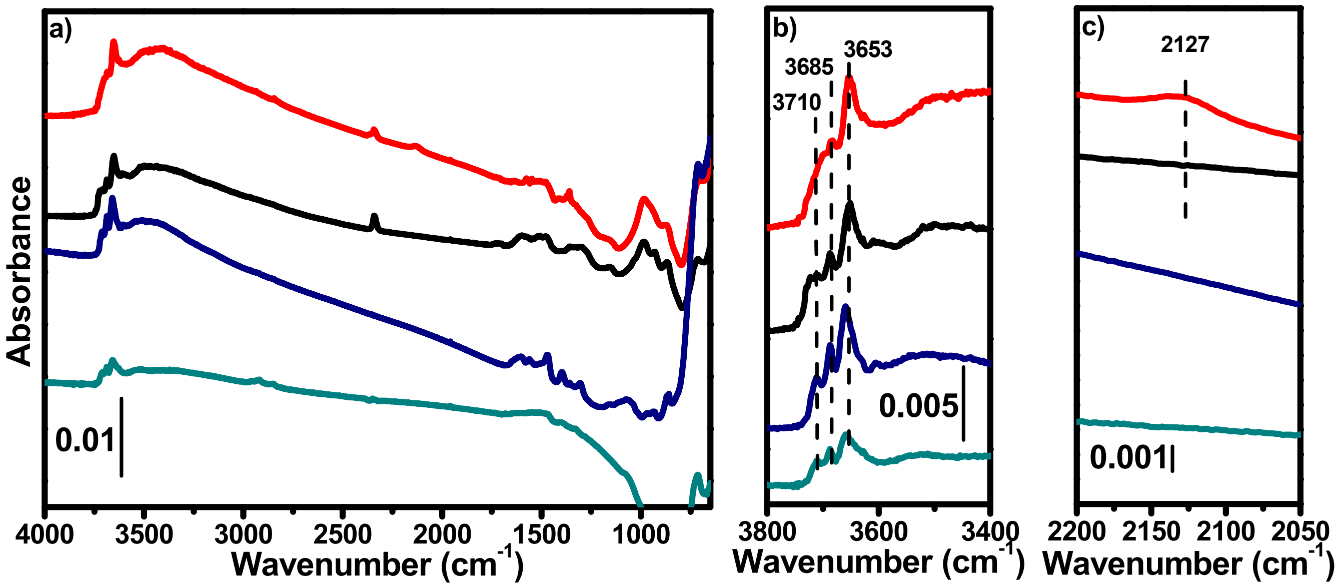

Figure S4. IR spectra of conv(650) (dark cyan line), MW(650) (blue line), MW(100) (black line) and MW(100)-red(red line) reported in the a) full spectrum region, b) ν(OH) region and c) Ce^{3+} ${}^2F_{5/2} \rightarrow {}^2F_{7/2}$ electronic transition region. Spectra have been collected at room temperature.

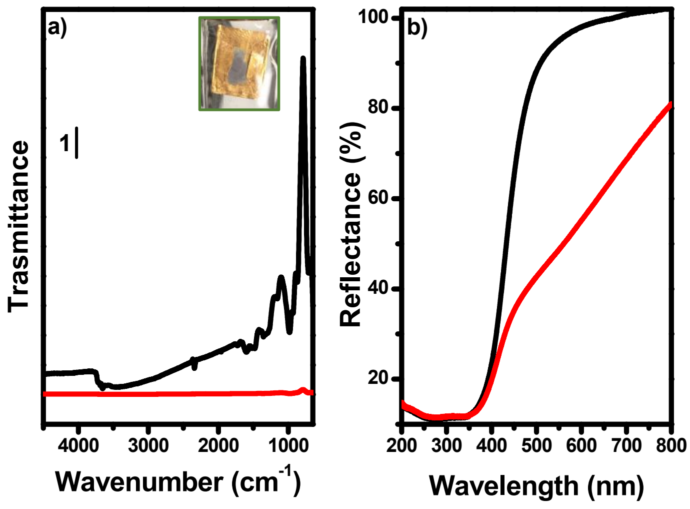

Figure S5. MW(100) (black line) and MW(100)-red (red line) a) FTIR transmittance and b) DRS UV-Vis spectra. A picture of MW(100)-red pellet used in the IR experiment is reported in the inset showing the dark colour of the sample.

To verify MW(100) structural integrity after reduction at 150°C, MW(100)-red PXRD pattern was measured from a sealed capillary and compared with MW(100). As shown in Figure S6, there is not

a clear difference between the two diffractograms, confirming as the reduction process influences the catalyst surface without modifying its structure and crystallites size.

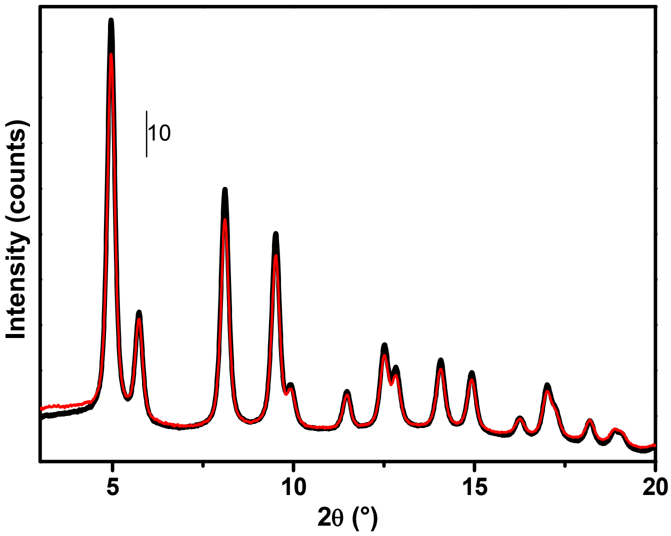

Figure S6. PXRD pattern of MW(100) (black line) and MW(100)-red (red line) collected in transmission mode with 46 keV monochromatic radiation at BM32 beamline of ESRF.

2.1 XPS beam damage evaluation and spectra fitting results

Beam damage of as-prepared MW(100) was evaluated for I) beam exposure during time and II) sample heating under vacuum. Considering dual anode beam damage (Figure S7a,b) we observed as by collecting spectra with 5min/scan (Figure S7a) the $Ce^{4+/3+}$ regions begin to vary after 30'. The first scan (5') presented Ce^{3+} abundance of 11% whilst averaging the 6 spectra in Figure S7a led to Ce^{3+}≈14%. Since there is not a considerable difference between the two spectra and fit results, we proceeded by collecting spectra with 30' time/scan for 330', observing as Ce^{3+} concentration increased to 15% after 60' of exposure while the sample temperature increased to 25°C. Eventually, Ce^{3+} concentration increased up to 25% after 330' exposure and temperature grew up to 35°C. On the contrary, catalyst damage induced by temperature under UHV (Figure S7b,c) showed a drastic increase of Ce^{3+} concentration already at 150°C, reaching values >45% at 400°C. These results showed as: I) dual anode exposure increased Ce^{3+} abundance of 4% in the first 60' and II) heating under vacuum had a stronger effect on Ce^{3+} oxidation state. Since at 50°C we observed 27% Ce^{3+} and after 330' of sample exposure to dual anode its temperature/Ce^{3+} reached 32°C/23%, we can conclude that heating from the X-Ray beam caused most of the damage from its exposure. To minimize the beam exposure effect on Ce(3d) region and following the evidence in Figure S7a, we then measured this region as the first one limiting the measurement to 30' time/scan.

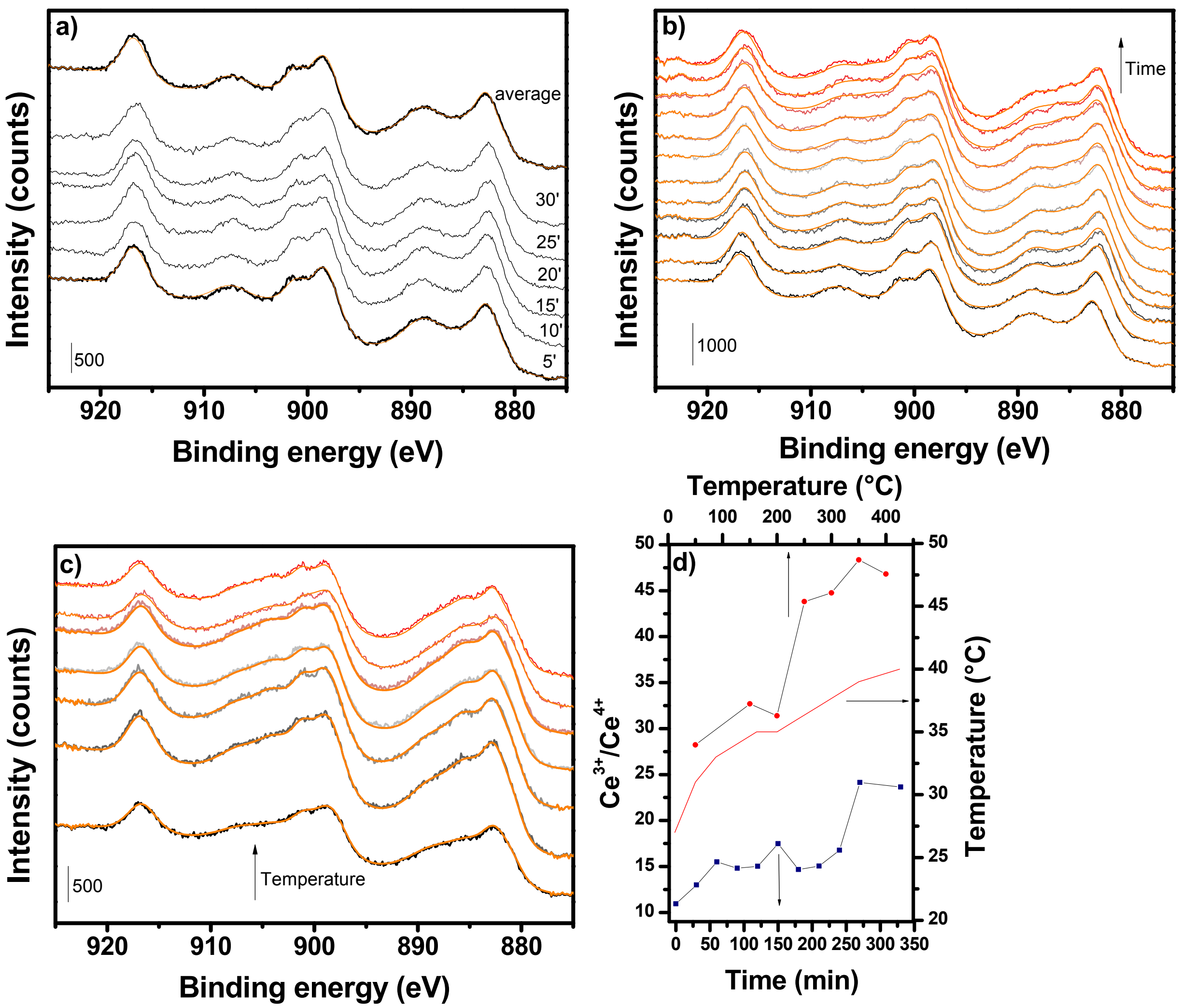

Figure S7. XPS experimental data and best fit curves (orange lines) collected : a) with 5’ time/scan for 30’ exposure to dual anode beam, b) with 30’ time/scan for 330’ exposure to dual anode beam (time increases from black to red line) and c) with 30’ time/scan during heating under UHV (temperature increases from black to red line). d) Ce^{+3}/Ce^{+4}concentration evaluated by best-fit curves during the time and temperature measurements. Samples stage temperature profile evolution during time experiment is reported with red line.

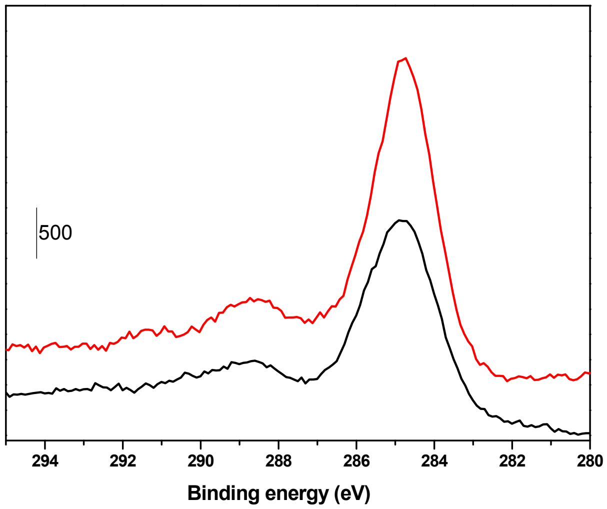

Figure S8. C 1s spectra of MW(100) (black line) and MW(100)-red (red line).

Table S3. XPS fitting results for MW(100) and MW(100)-red.

Sample		*v⁰*	*v*	*v'*	*v''*	*v'''*	*u⁰*	*u*	*u'*	*u''*	*u'''*
**MW(100)**	**BE(eV)**	880.7	883	885	888.9	898	899	901	904	907	916.7
	**FWHM (eV)**	1.83	2.81	3.59	4.39	2.86	1.83	2.81	3.59	4.39	2.86
	**Area**	999	42906	19849	37481	44442	669	28747	13299	25112	29776
**MW(100)-red**	**BE(eV)**	880.5	882.3	884.9	888.7	898	899	900.8	903	907.2	916.7
	**FWHM (eV)**	1.92	1.97	3.97	4.97	2.39	1.92	1.97	3.97	4.97	2.39
	**Area**	6908	22886	36052	24668	27562	4628	17271	24155	19077	19698

2.2 CO, CO_2 and CH_3OH adsorption investigated by FTIR

2.2.1 CO adsorption

The frequency position of CO interacting with Lewis acid sites strongly depends on the σ-donation/π-backdonation balance. The former reduces C≡O antibonding character, strengthening CO bond with consequent ν(CO) blueshift respect to the physisorbed CO frequency (2137 cm^{-1}) whilst the latter weakens CO bond by increasing its antibonding character hence causing ν(CO) redshift. On CeO_2 surface, three main scenarios can be observed I) Lewis acid sites with empty d/f orbitals i.e., Ce^{4+}=[Xe]$4f^05d^06s^0$, can accept an electron from the occupied CO σ antibonding orbitals (σ donation), II) atoms with partially filled d/f orbitals i.e., Ce^{3+}=[Xe] $4f^15d^06s^0$, parallelly to the first interaction can also donate an electron to CO unoccupied π orbitals (π-backdonation) and III) hydroxyl groups (Ce-OH) forming Bronsted acid sites, expected to interact with CO giving ν(CO)≈2153 cm^{-1} .[33] The latter interaction should perturb the ν(OH) vibration shifting it to lower and increasing its broadening. As observable in Figure S9, none of these effects were observed, hence

confirming the absence of an interaction between CO and hydroxyl groups indicating as they might behave as weak bases rather than acid sites.

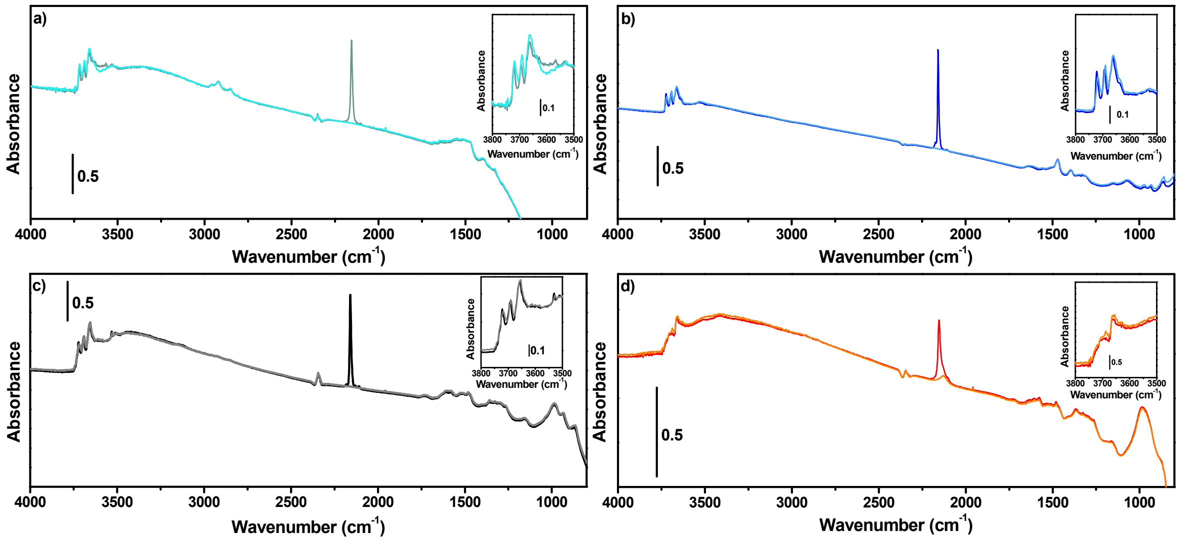

Figure S9. FTIR spectra of a) conv(650) , b) MW(650), c) MW(100) and d) MW(100)-red before and after interaction with CO at nominal 100K represented in lighter and darked colours, respectively.

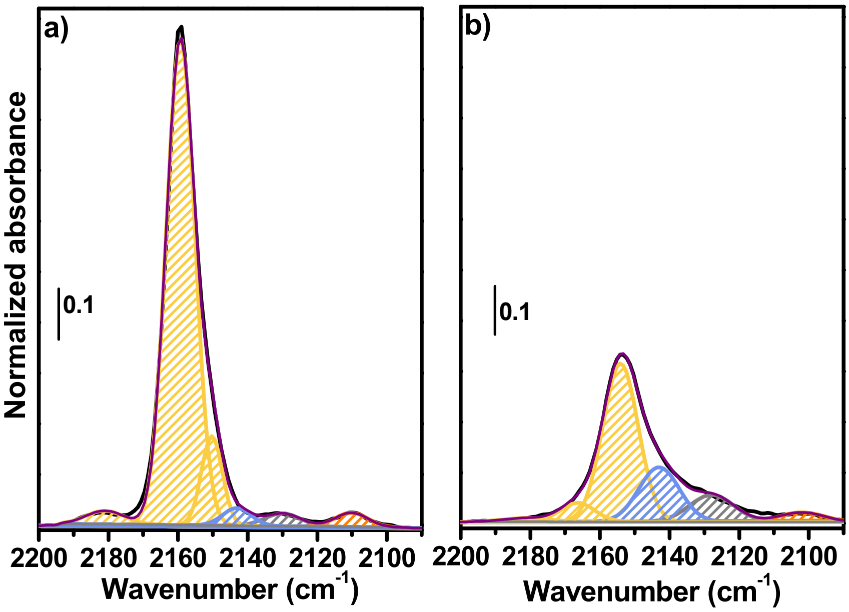

Figure S10. Experimental spectra (black line) and best fit (red line) of CO maximum coverage at LNT over: a)MW(100) and b)MW(100)-red. CO-Ce^{+4}, CO-Ce^{+3}, liquid-like CO and ^{13}CO-Ce^{+4} are indicated with yellow, grey, light blue and orange bands, respectively.

2.2.2 CH_3OH adsorption

Methanol is well known to be adsorbed over CeO_2 surface by reacting with its hydroxyl groups forming methoxide species represented in Figure S11 [4,5]

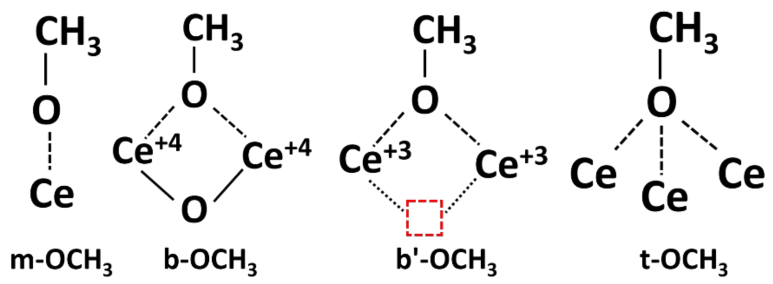

Figure S11. Sketched representation of potential methoxide species formed over CeO_2.

Figure S12 report the effect of 3mbar of methanol on the four materials under investigation followed by FTIR spectroscopy. In all cases hydroxyl groups were eroded however, their disappearance is not a proof of the formation of methoxides groups. Similarly, the ν(C-H) region was not so informative, since C-H stretching mode is less influenced from the support electronic state. The formation of methoxides is evident by the inspection of the ν(O-C) region, that allowed us to distinguish the different methoxide species formed. Monodentate (m-OCH_3), bi-bridged (b-OCH_3) and tri-bridged (t-OCH_3) methoxide groups (Figure S11) were observed over all the samples at 1109 cm^{-1}, 1058 cm^{-1} and 1033 cm^{-1}, respectively showing overall similar spectra for conv(650)/MW(650)/MW(100) catalysts. [4,5] Conv(650) presented such a weaker interaction with CH_3OH that a sharp band from gas phase-like methanol identified from the typical P, Q and R branches, can be observed at 1030 cm^{-1}.

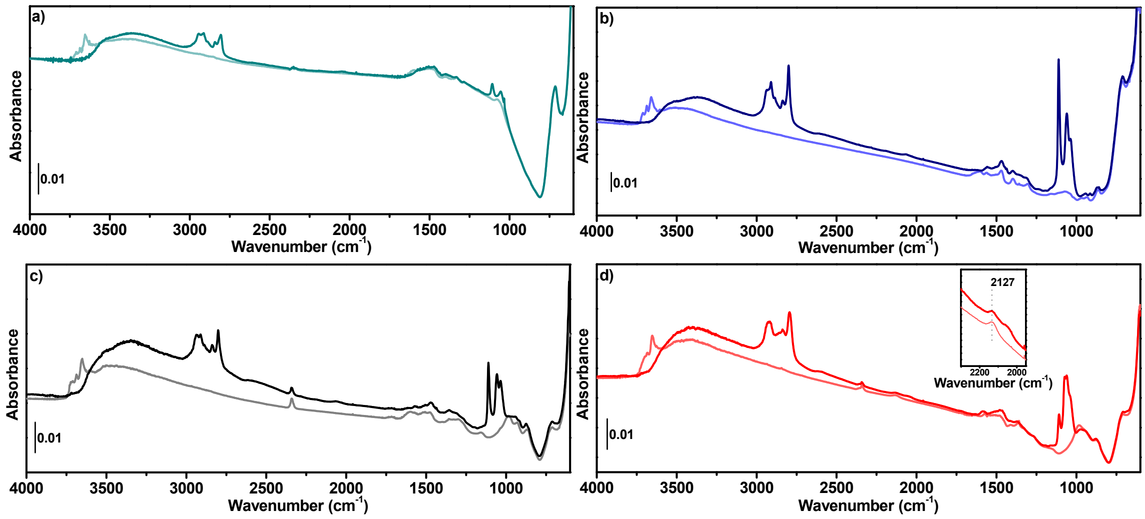

Figure S12. FTIR spectra of a) conv(650) , b) MW(650), c) MW(100) and d) MW(100)-red before and after interaction with CH_3OH (3mbar) at RT represented in lighter and darked colours, respectively.

2.2.3 CO_2 adsorption

Carbon dioxide is commonly adsorbed and activated over CeO_2 surface through formation of several carbonates (mono/bidentate/bridged), bicarbonates (bridged and bidentate) and formate (bridged and bidentate) species. As showed in the schematic representation in Figure S13 bicarbonates and formates can be easily distinguished from carbonates by the presence of vibrations related to -COH and -CH groups, respectively. Whilst a preliminary distinction between these three families is then straightforward, a fine identification of the precise formed member (mono/bi/tri-dentate and bridged) is not trivial. After CO_2 adsorption over all the materials (Figure S14 and Figure S15) we observed formation of two bands in the OH/CH stretching region (Figure S14a and Figure S15a) and six distinguishable bands in the carbonyls stretching regions (Figure S15c). The former region contains two bands at 3618 and at 2860 cm^{-1}, undoubtedly associated to ν(OH) and ν(CH) from bicarbonates and formate species, respectively, the latter being absent/too weak to be detected on conv(650).[3,6] On the contrary in the low energy region, due to their frequency position and separation, bands at 1577 and 1293 cm^{-1} can be ascribed to asymmetric and symmetric ν(CO) vibration, respectively, from carbonate species. The band at 1217 cm^{-1} is well reported as related to bicarbonates δ(COH), leading us to associate the band at 1401 cm^{-1} to ν(CO) from the same bicarbonate. A less distinguishable shoulder and a weak band, observed around 1604 and 1512 cm^{-1}, have been assigned after $^{13}CO_2$ adsorption (vide infra), to ν(CO) of bicarbonate and formate species, respectively.

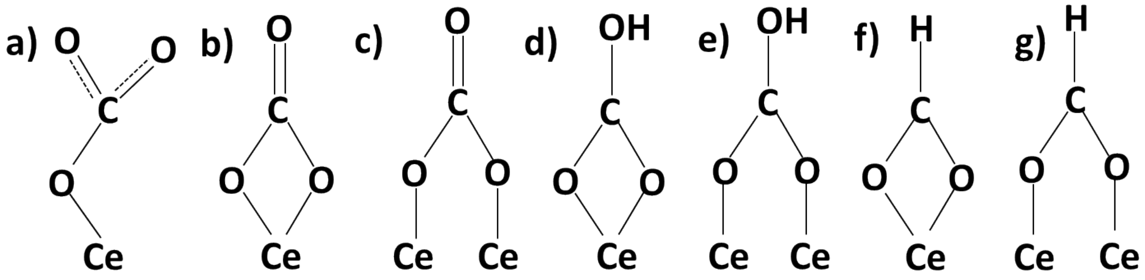

Figure S13. Schematic representation of a) monodentate carbonate, b) bidentate carbonate, c)bridged carbonate, d) bidentate bicarbonate, e) bridged bicarbonate, f) bidentate formate and g) bridged formate.

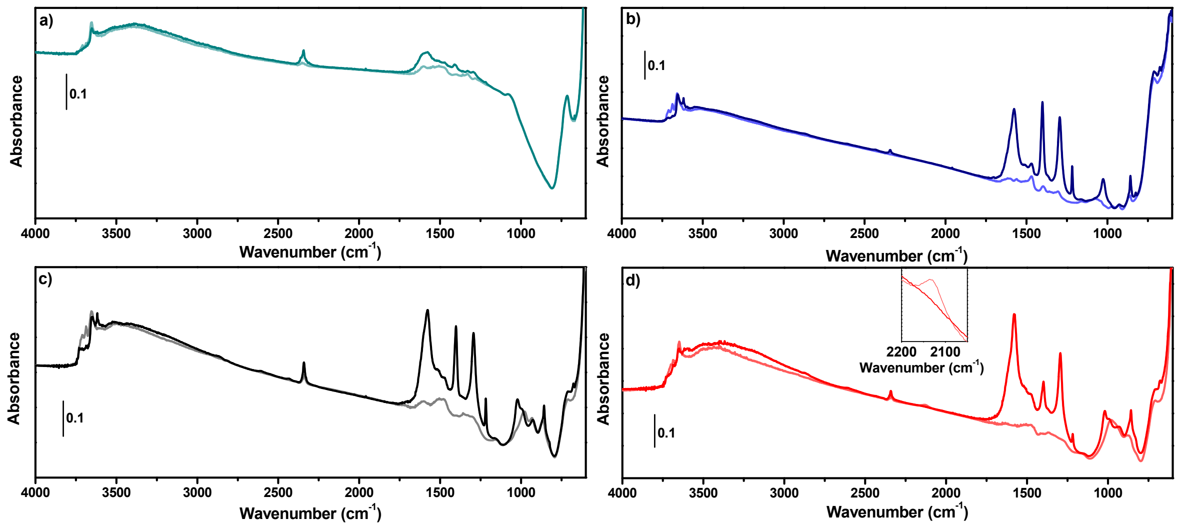

Figure S14. FTIR spectra of a) conv(650), b) MW(650), c) MW(100) and d) MW(100)-red before and after ineteraction with CO_2 (100 mbar) at RT represented in lighter and darked colours, respectively. Ce^{3+} electronic transition is reported in the inset in panel d.

Indeed, to finely assign mono/bidentate/bridged carbonates/bicarbonates vibrations, we performed $^{13}CO_2$ adsorption and compared the relative frequency shift with recent literature.[7] Following Vaysillov et al.[7] carbonates nomenclature, based on the number of cerium ions bounding each carbonates oxygen atom,$^{13}CO_2$ results (and TableS4) showed as the formed carbonates can be restricted to four bidentate species sketched in the main text (Figure S5) for clarity. Noteworthy, upon CO_2 adsorption over MW(100)-red we observed consumption of Ce^{3+} electronic transition at 2127 cm^{-1} suggesting a Ce^{3+}/CO_2 electronic interaction and a higher carbonates-to-bicarbonates ratio

respect to the other catalysts indicating, as rationalised hereafter, an increase of 1.21’ carbonate specie.

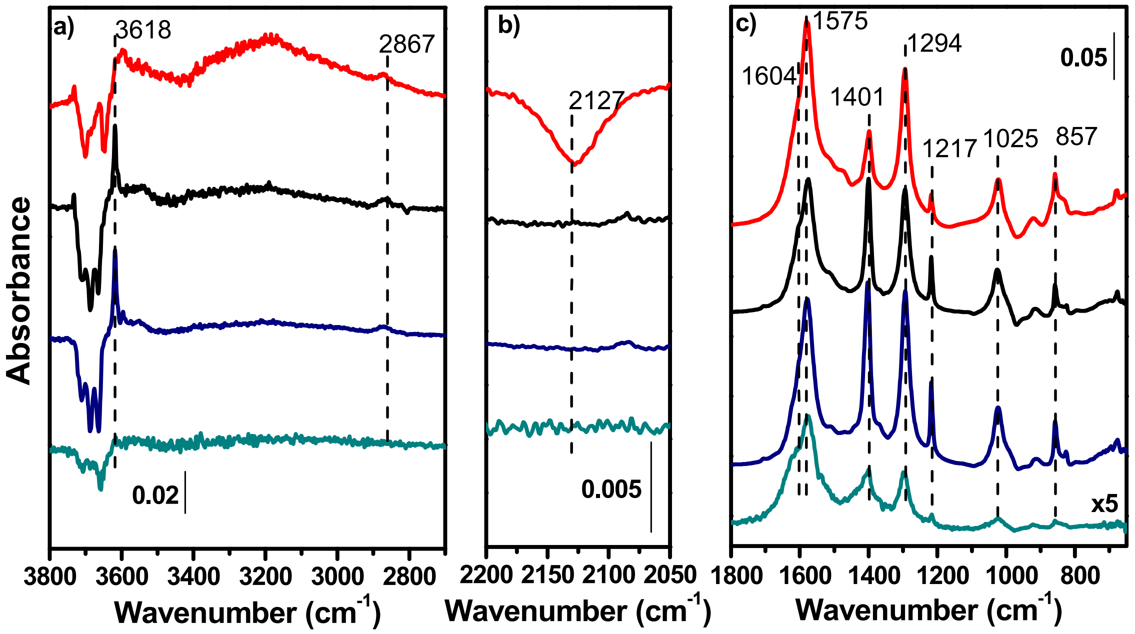

Figure S15. Difference FTIR spectra of 100 mbar CO_2 RT adsorption over, conv(650) (light blue line), MW(650) (dark blue line), MW(100) (black line) and MW(100)-red (red line) in the a) ν(OH) and ν(CH), b) Ce^{+3} electronic transition and c) ν(CO) spectral regions. Spectra are reported as difference by substracting the activated spectra showed in Figure S4a.

^{13}C shift allowed us to separate: I) a shoulder at 1604 cm^{-1} which position and chemical shift (Table S4) can be assigned to h-CO_3^- ν(CO) vibration and II) two weak bands at 1512 and 1365 cm^{-1} related to formate $\nu(CO)_{as/sym}$. On the contrary, bands at lower energy can be parallelly associate to $CO_3^=$/HCO_3 and HCO_2 i.e., i) band at 857 cm^{-1} matches the ^{13}C shift (Figure S16 and Table S4) of all the species and ii) band around 1025 cm^{-1} is considerably broad leading to an isotopic shift between 1 and 19 cm^{-1}, ascribable again to all the three mentioned species.

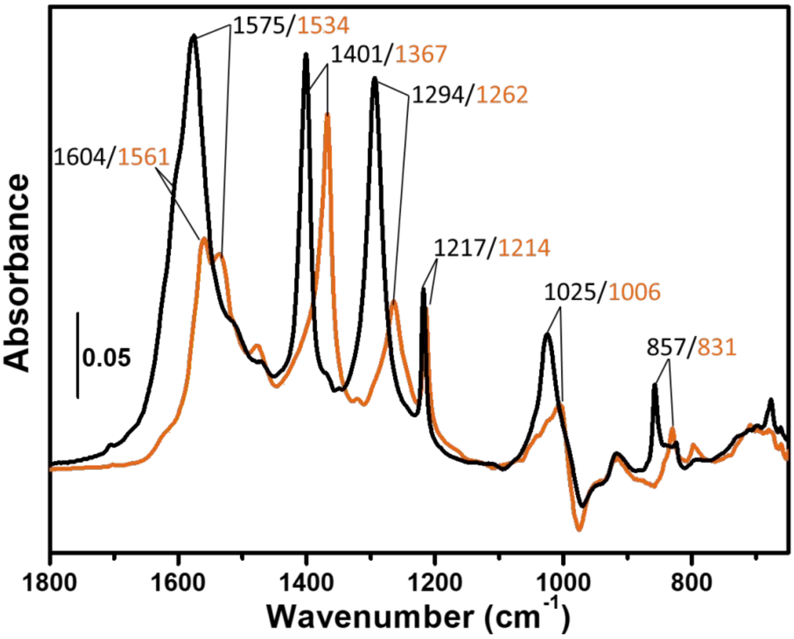

Figure S16. FTIR spectra of MW(100) after absorption of CO_2 (black line) and $^{13}CO_2$ (orange line). Main identified vibrations are indicated. Spectra are reported as difference by substracting the activated spectra.

Table S4. Experimental[a] and reference[b] vibrational frequencies (in cm^{-1}) from Vayssillov et al. [7], of carbonates, hydrogen carbonates and formate species observed over CeO_2 samples.

	**frequencies**					**Δ^{13}C**				
	ν(CO)	ν(CO)	ν(CO)	δ(OCO)		ν(CO)	ν(CO)	ν(CO)	δ(OCO)	
**$CO_3^=$ [a]**	1575	1294	1025	857		41	32	1-19	26	
**1.21[b]**	1492	1258	1005	823		41	29	5	26	
**1.2.1' [b]**	1503	1275	997	814		42	32	2	24	
**1.21' [b]**	1508	1297	1038	806		42	34	1	24	
**1.2.1 [b]**	1514	1258	990	818		42	30	4	25	
**1.2.1 [b]**	1536	1259	992	807		43	29	5	24	
[b]	ν(OH)	ν(CO)	δ(COH)	δ(OCO)		ν(OH)	ν(CO)	δ(COH)	δ(OCO)	
**h-CO_3^- [a]**	3618	1604	1218	1020		43	34	3	1-19	
[b]	3689	1588	1174	1000		42	35	1	4	
[b]	ν(CH)	ν(CO)	δ(COH)	ν(CO)	δ(COH)	ν(CH)	ν(CO)	δ(COH)	ν(CO)	δ(COH)
**formate [a]**	2860	1512	1365	1365	1020		36	45	45	1-19
[b]	2932	1553	1354	1346	1015	19	42	7	14	14

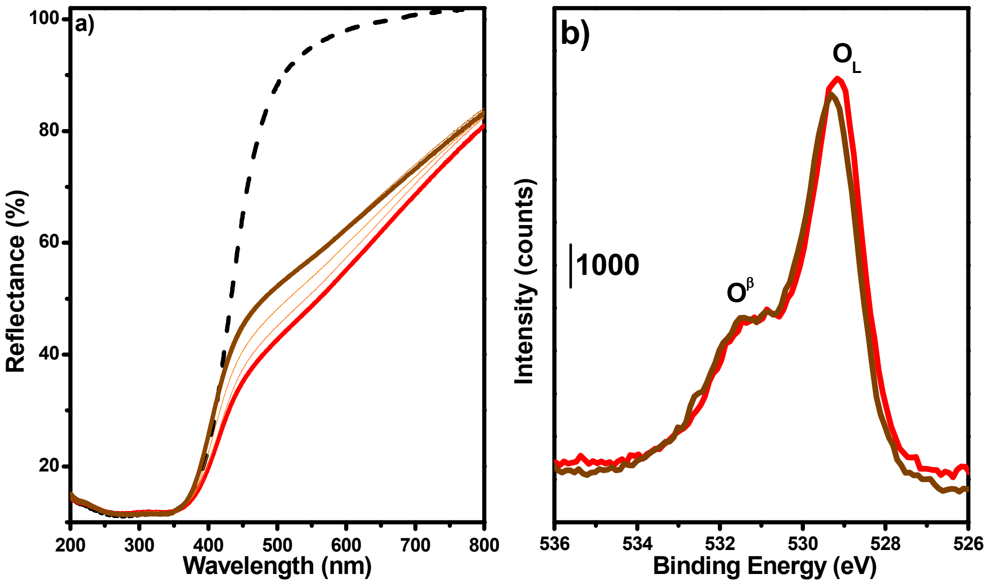

Figure S17. a) Ex-situ UV-Vis spectra of MW(100)-red prior (red line) and after interaction with CO_2 atmosphere. The effect of increasing CO_2 partial pressure from 25 to 100 mbar is illustrated by the spectra from red to brown line. MW(100) is showed with dashed black line. b) XPS O 1s experimental spectra of MW(100)-red before (red line) and after interaction with 100 mbar of CO_2 (brown line)

2.2.4 CH_3OH adsorption over CeO_2 previously saturated with CO_2 (CO_3-CeO_2)

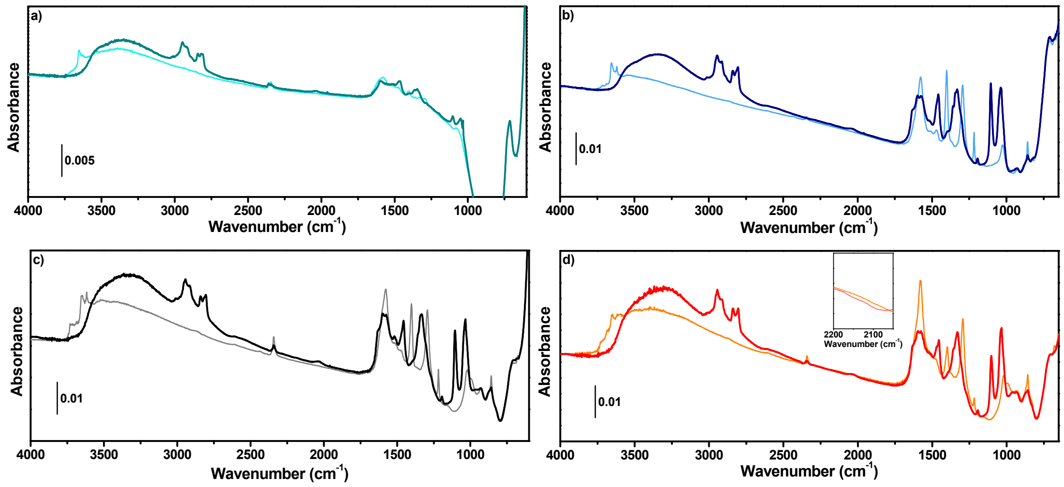

Figure S18. FTIR spectra of a) conv(650), b) MW(650), c) MW(100) and d) MW(100)-red after interaction with 100 mbar CO_2 (lighter colour lines) followed by 3 mbar of CH_3OH (darker colour lines) at RT. Ce^{3+} electronic transition is showed in panel c inset.

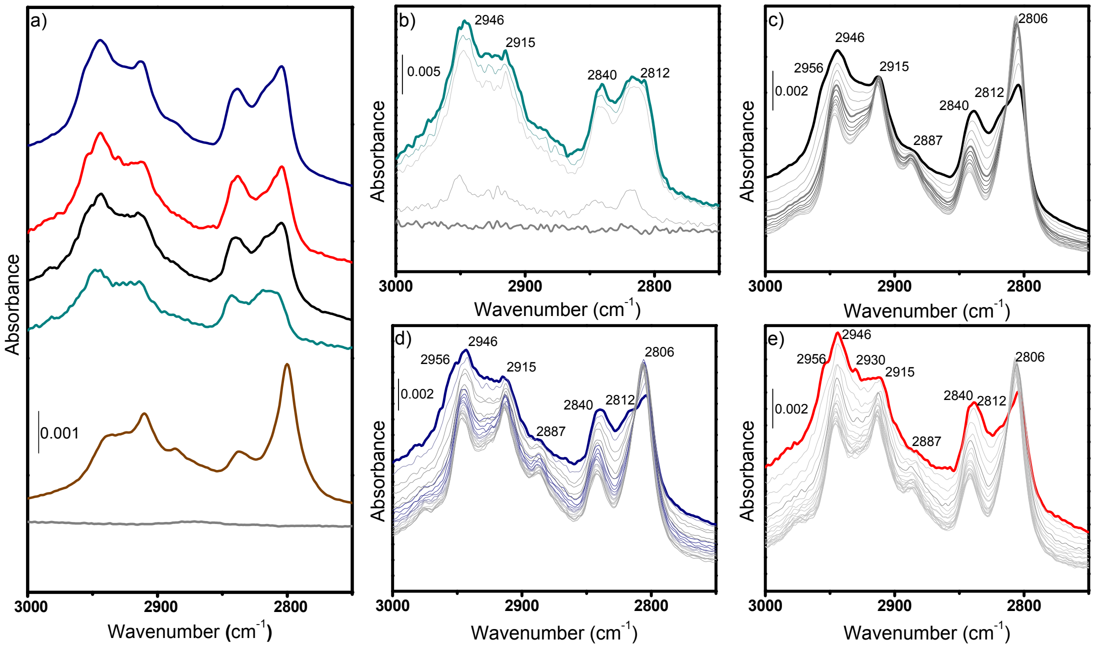

Figure S19 ν(CH) region detail of a) difference IR spectra of conv(650) (light blue line), MW(650) (blue line), MW(100) (dark line) and MW(100)-red (red line). b,c,d,e) FTIR spectra evolution of adsorption of methanol (3 mbar) over b) conv(650), c) MW(650), d) MW(100) and e) MW(100)-red previously exposed to 100 mbar of CO_2. Adsorption time evolution goes from grey line to coloured line. Spectra of each CO_3-CeO_2 component have been subtracted. CH_3O-MW(650) (grey line) and CO_3-MW(650) (brown line) component are showed for clarity.

Table S5 Infrared vibrational frequencies and their assignment.

Wavenumber (cm^{-1})	Vibration [species]	Ref.
1195	δ(OCO) [MMC]	8
1210	δ(COH) [hCO_3^-]	7
1295/1300	$\nu(CO)_{sym}$ [$CO_3^=$]	7
1335/1340	$\nu(OCO)_{sym}$ [MMC]	8
1360/1364	$\nu(OCO)_{sym}$ [$HCOO^-$]	7
1458/1464	$\nu(OCO)_{as}$ [MMC]	8
1569/1576	$\nu(CO)_{as}$ [$CO_3^=$]	7
1596/1600	ν(C=O) [MMC]	8
1630	$\nu(OCO)_{as}$ [$HCOO^-$]	7
2789	$\nu(CH_3)_{sym}$ [b’-CH_3O-]	11
2804	$\nu(CH_3)_{sym}$ [m/b/t-CH_3O-]	11
2812	$\nu(CH_3)_{as}$ [m-CH_3O-]	11
2838	ν(CH) [$HCOO^-$]	7
2887	2δ(CH_3) [CH_3O-]/ ν(CH_3) [MMC]	11
2912	$\nu(CH_3)_{as}$ [b-CH_3O-]	11
2942	ν(CH) [$HCOO^-$]	7
2956	ν(CH_3) [MMC]	8

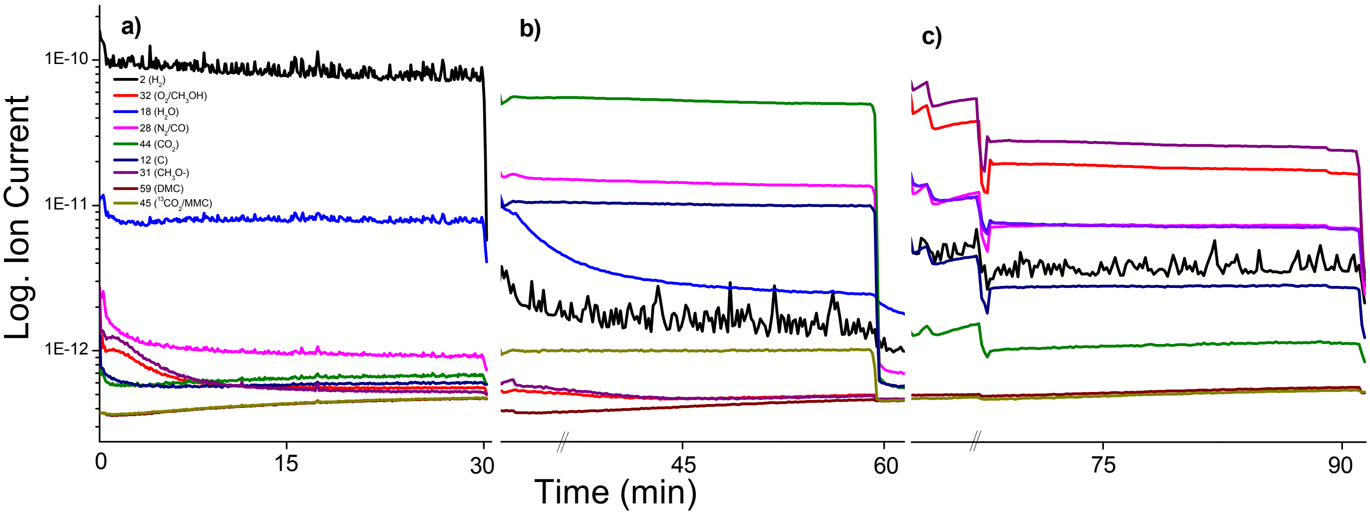

Figure S20. Mass-Spectra signals of masses 2(black line), 32 (red line), 18 (light blue line), 28 (magenta line), 44 (green line), 12 (dark blue line), 31 (purple line), 59 (brown line) and 45 (dark yellow) measured during MW(100) a)150°C H_2 treatment followed by b) RT CO_2 adsorption and c) RT CH_3OH adsorption.

2.2.5 CO_2 adsorption over CeO_2 previously saturated with CH_3OH (CH_3O-CeO_2)

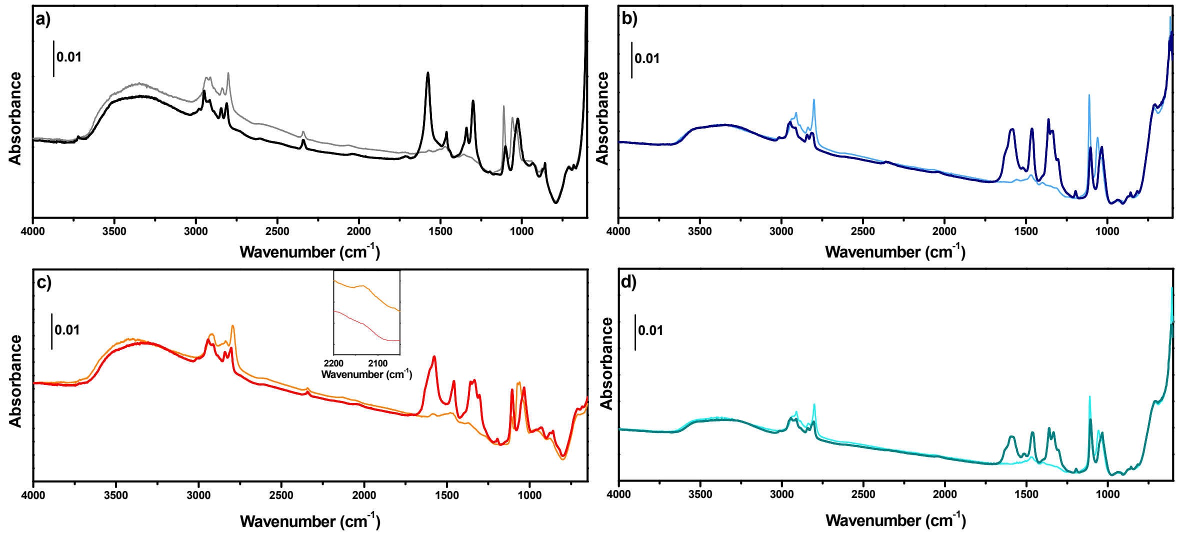

Figure S21. FTIR spectra of a) MW(100) , b) MW(650), c)MW(100)-red and d) conv(650) after interaction with 3 mbar CH_3OH (lighter colour lines) followed by 100 mbar of CO_2 (darker colour lines) at RT. Ce^{+3} electronic transition is showed in panel c inset.

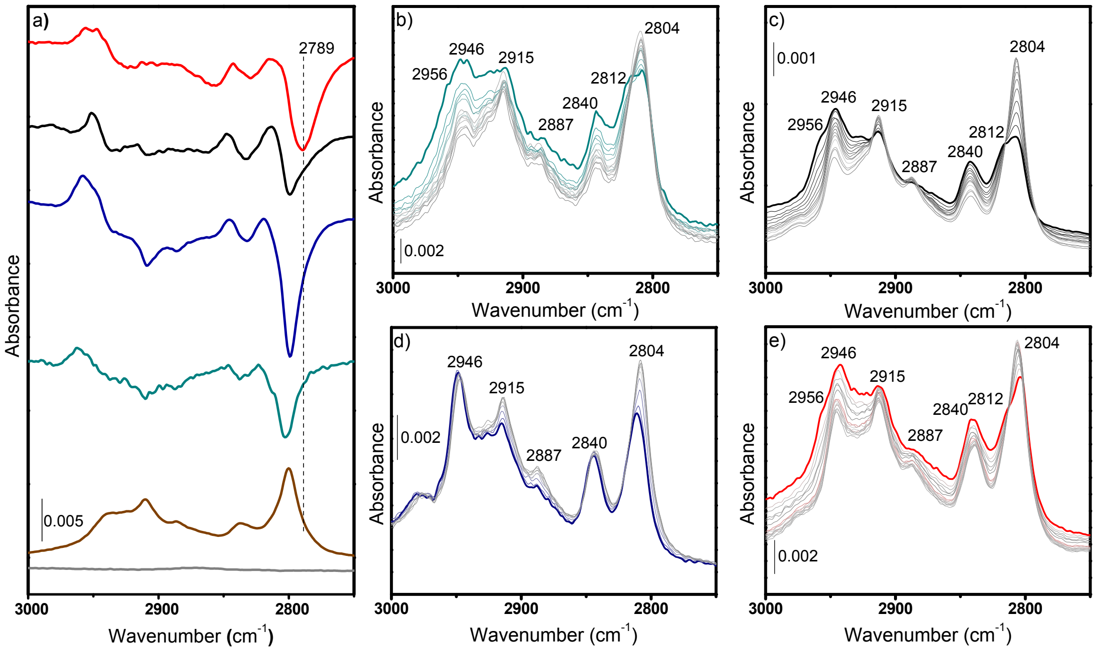

D

Figure S22 ν(CH) region detail of a) difference IR spectra of conv(650) (light blue line), MW(650) (blue line), MW(100) (dark line) and MW(100)-red (red line). b,c,d,e) FTIR spectra evolution of adsorption of CO_2 (100 mbar) over b) conv(650), c) MW(650), d) MW(100) and e) MW(100)-red previously exposed to 3 mbar of CH_3OH. Adsorption time evolution goes from grey line to coloured line. Spectra of each CH_3O-CeO_2 component have been subtracted. CH_3O-MW(650) (grey line) and CO_3-MW(650) (brown line) component are showed for clarity.

References

(1) Batool, T.; Bukhari, B. S.; Riaz, S.; Batoo, K. M.; Raslan, E. H.; Hadi, M.; Naseem, S. Microwave Assisted Sol-Gel Synthesis of Bioactive Zirconia Nanoparticles – Correlation of Strength and Structure. *J. Mech. Behav. Biomed. Mater.* **2020**, *112* (August).

(2) Badri, A.; Binet, C.; Lavalley, J. An FTIR Study of Surface Ceria Hydroxy Groups during a Redox Process with H2. **1996**, *92* (c), 4669–4673.

(3) Binet, C.; Daturi, M.; Lavalley, J. C. IR Study of Polycrystalline Ceria Properties in Oxidised and Reduced States. *Catal. Today* **1999**, *50* (2), 207–225.

(4) Binet, C.; Badri, A.; Lavalley, J. C. A Spectroscopic Characterization of the Reduction of Ceria from Electronic Transitions of Intrinsic Point Defects. *J. Phys. Chem.* **1994**, *98* (25), 6392–6398.

(5) Badri, A.; Binet, C.; Lavalley, J. C. Use of Methanol as an IR Molecular Probe to Study the Surface of Polycrystalline Ceria. *J. Chem. Soc. - Faraday Trans.* **1997**, *93* (6), 1159–1168.

(6) Li, C.; Sakata, Y.; Arai, T.; Domen, K.; Maruya, K.; Onishi, T. Adsorption of Carbon Monoxide and Carbon Dioxide on Cerium Oxide Studied by Fourier-Transform Infrared Spectroscopy. Part 2.—Formation of Formate Species on Partially Reduced CeO2 at Room

Temperature. *J. Chem. Soc. Faraday Trans. 1 Phys. Chem. Condens. Phases* **1989**, *85* (6), 1451–1461.

(7) Vayssilov, G. N.; Mihaylov, M.; Petkov, P. S.; Hadjiivanov, K. I.; Neyman, K. M. Reassignment of the Vibrational Spectra of Carbonates , Formates , and Related Surface Species on Ceria : A Combined Density Functional and Infrared Spectroscopy Investigation. *J. Phys. Chem. C* **2011**, *115* (47), 23435–23454.

(8) Taek Jung, K.; Bell, A. T. An in Situ Infrared Study of Dimethyl Carbonate Synthesis from Carbon Dioxide and Methanol over Zirconia. *J. Catal.* **2001**, *204* (2), 339–347.

(9) Katon, J. E.; Cohen, M. D. The Vibrational Spectra and Structure of Dimethyl Carbonate and Its Conformational Behavior. *Can. J. Chem.* **1975**, *53* (9), 1378–1386.

(10) Tomishige, K.; Ikeda, Y.; Sakaihori, T.; Fujimoto, K. Catalytic Properties and Structure of Zirconia Catalysts for Direct Synthesis of Dimethyl Carbonate from Methanol and Carbon Dioxide. *J. Catal.* **2000**, *192* (2), 355–362.

(11) Binet, C.; Daturi, M. Methanol as an IR Probe to Study the Reduction Process in Ceria-Zirconia Mixed Compounds. *Catal. Today* **2001**, *70* (1–3), 155–167.

(12) Aresta, M.; Dibenedetto, A.; Pastore, C.; Cuocci, C.; Aresta, B.; Cometa, S.; De Giglio, E. Cerium(IV)Oxide Modification by Inclusion of a Hetero-Atom: A Strategy for Producing Efficient and Robust Nano-Catalysts for Methanol Carboxylation. *Catal. Today* **2008**, *137* (1), 125–131.

(13) Aresta, M.; Dibenedetto, A.; Pastore, C.; Angelini, A.; Aresta, B.; Pápai, I. Influence of Al2O3 on the Performance of CeO2 Used as Catalyst in the Direct Carboxylation of Methanol to Dimethylcarbonate and the Elucidation of the Reaction Mechanism. *J. Catal.* **2010**, *269* (1), 44–52.
